# A prognostic NAD+ metabolism-related gene signature for predicting response to immune checkpoint inhibitor in glioma

**DOI:** 10.3389/fonc.2023.1051641

**Published:** 2023-02-08

**Authors:** Cheng Jiang, Yujie Zhou, Lizhao Yan, Jianglin Zheng, Xuan Wang, Junjun Li, Xiaobing Jiang

**Affiliations:** ^1^ Department of Neurosurgery, Union Hospital, Tongji Medical College, Huazhong University of Science and Technology, Wuhan, China; ^2^ Department of Hand Surgery, Wuhan Union Hospital, Tongji Medical College, Huazhong University of Science and Technology, Wuhan, China

**Keywords:** glioma, NAD+ metabolism, prognosis, immune checkpoint inhibitor, signature

## Abstract

**Background:**

Nicotinamide adenine dinucleotide (NAD+) metabolism is involved in a series of cancer pathogenesis processes, and is considered a promising therapeutic target for cancer treatment. However, a comprehensive analysis of NAD+ metabolism events on immune regulation and cancer survival has not yet been conducted. Here, we constructed a prognostic NAD+ metabolism-related gene signature (NMRGS) associated with immune checkpoint inhibitor (ICI) efficacy in glioma.

**Methods:**

40 NAD+ metabolism-related genes (NMRGs) were obtained from the Reactome database and the Kyoto Encyclopedia of Genes and Genomes (KEGG) database. Glioma cases with transcriptome data and clinical information were obtained from Chinese Glioma Genome Atlas (CGGA) and The Cancer Genome Atlas (TCGA). NMRGS was constructed based on the calculated risk score using univariate analysis, Kaplan–Meier analysis, multivariate Cox regression, and nomogram. This NMRGS was verified in training (CGGA693) and validation (TCGA and CGGA325) cohorts. The immune characteristics, mutation profile, and response to ICI therapy were subsequently analyzed for different NMRGS subgroups.

**Results:**

Six NAD+ metabolism-related genes, including CD38, nicotinamide adenine dinucleotide kinase (NADK), nicotinate phosphoribosyltransferase (NAPRT), nicotinamide/nicotinic acid mononucleotide adenylyltransferase 3 (NMNAT3), poly(ADP-Ribose) polymerase family member 6 (PARP6), and poly(ADP-Ribose) polymerase family member 9 (PARP9), were ultimately used to construct a comprehensive risk model for glioma patients. Patients in the NMRGS-high group showed a poorer survival outcome than those in the NMRGS-low group. The area under curve (AUC) indicated that NMRGS has good potential in glioma prognostic prediction. A nomogram with improved accuracy was established based on independent prognostic factors (NMRGS score, 1p19q codeletion status, and WHO grade). Furthermore, patients in the NMRGS-high group showed a more immunosuppressive microenvironment, higher tumor mutation burden (TMB), higher human leucocyte antigen (HLA) expression and a more therapeutic response to ICI therapy.

**Conclusions:**

This study constructed a prognostic NAD+ metabolism-related signature associated with the immune landscape in glioma, which can be used for guiding individualized ICI therapy.

## Introduction

Glioma is the most common primary brain tumor originating from neuroglial progenitor cells, accounting for 80% of malignant tumors in the central nervous system (1). According to the WHO grading system, gliomas are classified into four grades. Glioblastoma (GBM; WHO grade IV) is the most aggressive glioma type, with a median overall survival of approximately 15 months (2). Despite advances in surgical treatment, radiotherapy, and chemotherapy, the effectiveness of glioma treatment is still not satisfactory (3). In recent years, immunotherapy like immune checkpoint inhibitors (ICIs) has emerged as a promising treatment for glioma patients (4). However, given that the immune environment is frequently immunosuppressive and characterized by T cell exhaustion, M2 macrophage infiltration, and increased expression of immune checkpoints, immunotherapy for glioma still faces significant challenges. Overcoming tumor immune resistance to promote tumor eradication has become a problem for immunotherapy (5). On the basis of the inherent intra-tumor heterogeneity, individual tumors may contain immunologically distinct subtypes, each of which will respond differently to immunotherapy treatment. Thus, it is essential to identify molecular markers that accurately evaluate prognosis and guide individualized immunotherapy in glioma patients.

Metabolic reprogramming is recognized to occur in all cellular components of the tumor microenvironment. NAD+ is an essential redox cofactor in cellular metabolism, affecting gene expression, energy production, glycolysis, DNA repair, and cell cycle progression (6). Several processes associated with NAD+ signaling are dysregulated in cancer (7). High levels of NAD+ have been consistently observed in gliomas, and 90% of gliomas are sensitive to NAD+ depletion (8). Recently, several studies have indicated that NAD metabolism is involved in cancer immune suppression (9, 10). For example, nicotinamide phosphoribosyltransferase (NAMPT)-mediated NAD+ metabolism enhanced interferon gamma-induced programmed cell death 1 ligand 1 (PD-L1) expression and drove tumor immune evasion in a CD8+ T cell-dependent manner (11). Microparticle delivery of NAMPT inhibitor targeting NAD+ salvage pathway at the tumor site was found to alter an immune tumor microenvironment that could potentiate checkpoint immunotherapy for glioblastoma (12). The NAD-dependent deacetylase SIRT2 participated in tumor immune response by regulating T cell differentiation (13). Therefore, the immunotherapy strategy based on NAD+ metabolism reprogramming to achieve anti-cancer benefits is highly promising. However, current studies have mainly focused on the function of individual NMRG in only a few cases. A comprehensive analysis based on a large-scale cohort for glioma immunotherapy is needed.

In this study, we systematically profiled the NAD+ Metabolism-Related genes of glioma patients and then developed a prognostic signature for glioma. We then investigated the potential value of this signature in predicting prognosis, evaluating the immune microenvironment, and guiding clinical treatment in glioma patients. Our findings demonstrated that NMRGS score was a promising prognostic index associated with the therapeutic effect of ICI therapy in glioma.

## Materials and methods

### Acquisition of data in patients with glioma

The Cancer Genome Atlas (TCGA, https://portal.gdc.cancer.gov/) database and the Chinese Glioma Genome Atlas (CGGA, http://www.cgga.org.cn/) database were employed for downloading RNA-seq transcriptome data and clinical information of glioma patients. The criteria for including patients were as follows: (a) patients with survival data and overall survival (OS) ≥ 30 days; (b) patients with mRNA sequencing data; and (c) patients with definitive histopathological diagnosis. With these inclusion criteria, 1486 patients with gliomas were included in the follow-up analysis. Specifically, the CGGA693 dataset (n=638) served as a training cohort. At the same time, the TCGA dataset (n=550) and the CGGA325 dataset (n=298) served as a validation cohort. The detailed clinical information of glioma patients from the training and validation cohort was shown in **Supplementary Table S1**.

### Characteristics evaluation of the NAD+ metabolism-related genes

40 NAD+ Metabolism-Related genes were obtained from the Reactome database (R-HAS-196807) and Kyoto Encyclopedia of Genes and Genomes database (has00760). The detail of the NAD+ Metabolism-Related genes is shown in (**Supplementary Table S2**). Pearson correlation analysis among the NAD+ Metabolism-Related genes was conducted in the CGGA693 set. RNA-seq transcriptome data of normal tissue were derived from Genotype-Tissue Expression (GTEx, https://www.gtexportal.org/) database. The “clusterProfiler” software package of R (version 4.1.3) was used to analyze the gene ontology (GO) and KEGG pathway for functional annotation of these NAD+ metabolism-related genes with a significant p-value (<0.05).

### Construction and validation of the prognostic NMRGS

Univariate Cox regression analysis was performed to identify OS-associated NMRGs (p < 0.05). Kaplan-Meier (K-M) method was used further prognostic identification (p < 0.05). Subsequently, the multivariate Cox regression analysis was then used to filter the independent OS-associated NMRGs. The Lasso regression analysis was conducted to ensure that overfitting was avoided. Finally, the selected NMRGs were applied to establish the NMRGS based on the multivariate Cox regression coefficient. The risk score for each patient was determined using Equation: NMRGS score = expression level of gene_1_ × coefficient of gene_1_ + expression level of gene_2_ × coefficient of gene_2_ +… + expression level of gene_n_ ×coefficient of gene_n_.

Patients were then divided into two groups according to the median NMRGS score, including the NMRGS-low group and the NMRGS-high group. The difference in clinicopathologic factors and NMRGs expression between the NMRGS-low group and the NMRGS-high group was exhibited with a heatmap. The comparison of OS between the NMRGS-low group and the NMRGS-high group was conducted using K-M survival curves with the “survminer” package in R. The receiver operating characteristic (ROC) curves were plotted to evaluate the accuracy of NMRGS with the “timeROC” package in R. Univariate and multivariate Cox regression analyses were conducted to identify the NMRGS score as an independent predictor for OS with the “survival” package in R. Similar validation analyses were performed simultaneously in both training and validation cohorts.

To predict the one-, three-, and five-year survival rates of glioma patients, we constructed a nomogram based on NMRGS score and the independent prognostic clinicopathologic parameters in the training cohort by employing the R package “rms,” “regplot,” and “Hmisc. The availability of this nomogram was evaluated by the C-indices and calibration curves.

### Mutation profile analysis

TMB is defined as the total number of somatic mutations and has emerged as a quantitative biological marker of the immune response. We analyzed the TMB value and visualized the mutation profiles in the NMRGS-low group and the NMRGS-high group *via* the “Maftools” package in R. Relevant mutation data were downloaded from the TCGA database. The correlation between TMB value and NMRGS score was analyzed with the spearman method. The influence of TMB value on glioma patient survival was evaluated among the NMRGS-low group and the NMRGS-high group with K-M method.

### Tumor microenvironment (TME) analysis and ICI therapy response

Enrichment analysis to understand the immune-associated signal transduction pathways in which the NMRGS were involved was conducted using Gene set enrichment analysis (GSEA) between the NMRGS-low group and the NMRGS-high group. Those signaling pathways meeting the screening criterion (P < 0.05 and false discovery rate (FDR) < 0.25) were considered significantly enriched. Single-sample GSEA (ssGSEA) analysis was conducted to identify immune cell-related infiltrating scores and immune-related pathways using the “gsva” package in R. In addition, CIBERSORT was used to determine the relative proportions of 22 types of immune cells between the NMRGS-low group and the NMRGS-high group. ImmuneScore, StromalScore, ESTIMATEScore, and TumorPurity were calculated by ESTIMATE (Estimation of Stromal and Immune Cells in Malignant Tumor Tissues Using Expression Data) algorithm utilizing the R-package “estimate.” The relationships between NMRGS score and the recognized immune checkpoint genes were conducted. In addition, Tumor immune dysfunction and exclusion (TIDE, https://tide.dfci.harvar.edu) algorithm were used to assess the difference in sensitivity to immunotherapy between the NMRGS-low group and NMRGS-high group.

### Clinical sample collection and expression detection

All clinical samples obtained by surgical resection from glioma patients were collected from the Neurosurgery Department of Wuhan Union Hospital. The informed consent was acquired from each involved patient. In order to detect the expression of NMRGs at mRNA level in tissues, total RNA was extracted by RNAiso Plus (Takara 9109) and reverse transcribed into cDNA with RT Supermix Reagent Kit (Vazyme R323). The detection and amplification of the respective genes were conducted using SYBR qPCR Master Mix (Q311-02/03) according to the manufacturer instruction. Correlation data was calculated based on the ΔΔCT of the target gene, and Glyceraldehyde-3-Phosphate Dehydrogenase (GAPDH) was kept as an endogenous control. The primer sequences are shown in **Supplementary Table S3**. Tissue samples were then investigated for NMRGs expression by immunohistochemistry. All specimens were sectioned four μm thick after being fixed with 10% formalin and embedded in paraffin. Sections were deparaffinized in xylene and then hydrated with a descending alcohol concentration. After dewaxing and hydration, the sections were boiled in citrate buffer (pH=6) to realize antigen retrieval and treated with methanol containing 3% hydrogen peroxide to inhibit endogenous peroxidase activity. To block nonspecific staining, tissue sections were incubated with 3% bovine serum albumin. Next, the slides were set at 4°C overnight with a primary antibody for NMRGs. Secondary antibodies were added for incubation at 37°C for 50 min after washing with PBS. After visualization with diaminobenzidine, sections were counterstained with hematoxylin, followed by cover glasses mounting. The details of primary antibodies are shown in **Supplementary Table S4**.

### Statistical analysis

R version 4.1.3(Institute of Statistics and Mathematics, Vienna, Austria), Perl language, and GraphPad Prism 8.3.0 (GraphPad Software Inc, La Jolla, CA, USA) were used to analyze the data or visualized pictures. Mann–Whitney U-test was used to detect the differences in the continuous variables between the NMRGS-low group and the NMRGS-high group. Comparisons of categorical variables between the NMRGS-low group and the NMRGS-high group were executed with the Chi-square test. Spearman’s test was used to evaluate the correlation coefficient. P value <0.05 was regarded as statistically significant (*P < 0.05, **P < 0.01, ***P < 0.001).

## Results

### NMRGs is differentially expressed in glioma tissues compared with normal brain tissue and is correlated with glioma grade

**Figure 1:**
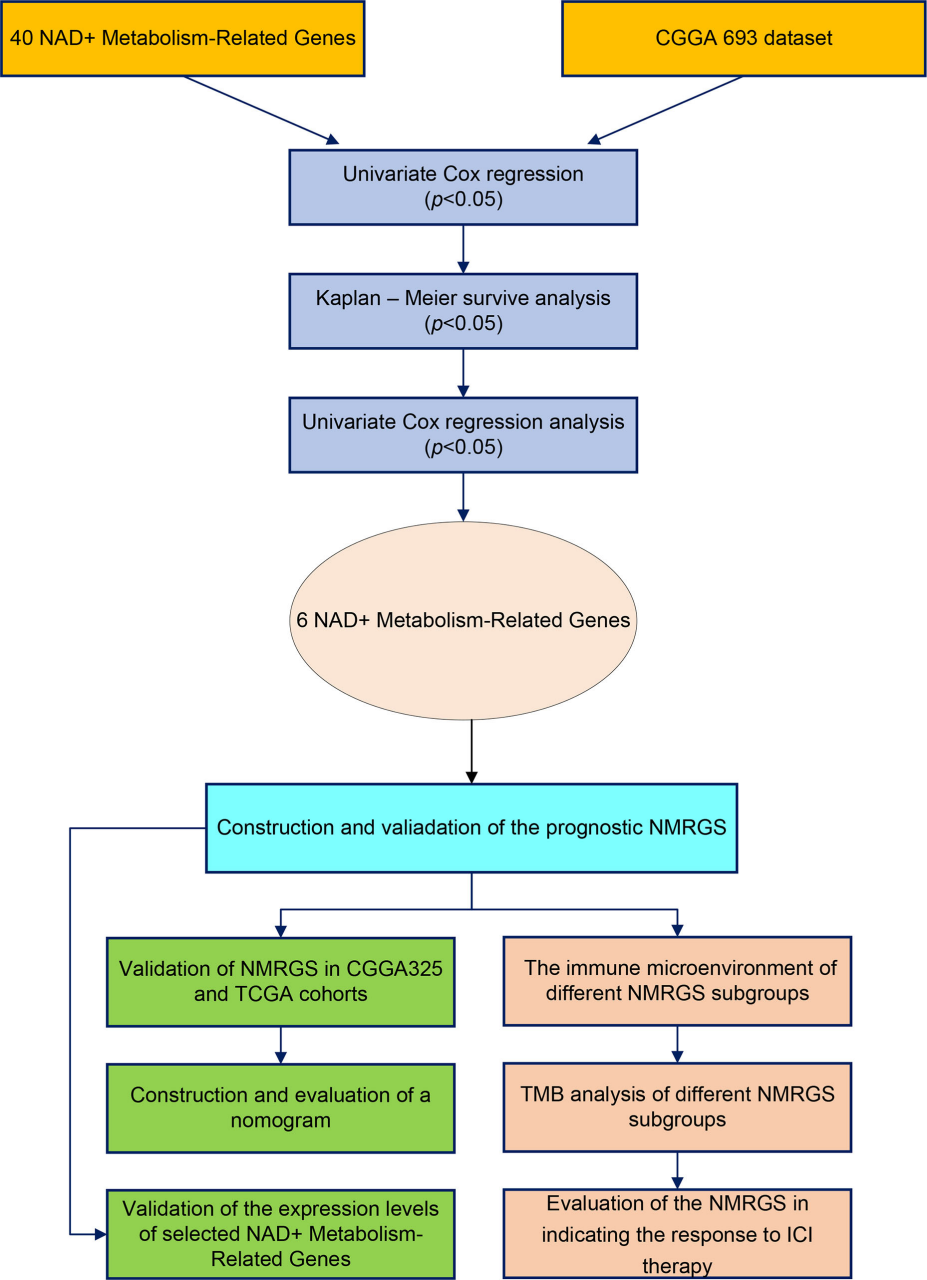
Flow chart of this study.

**Figure 2:**
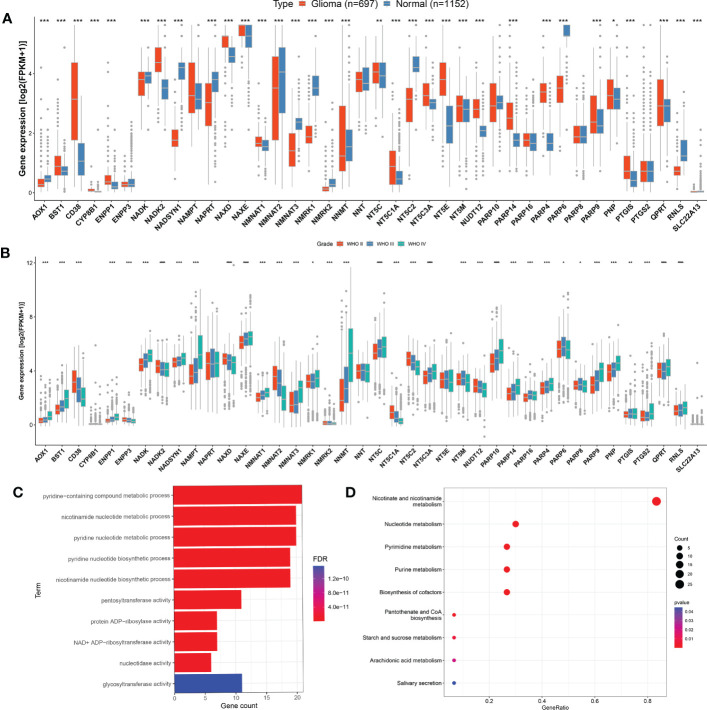
Evaluation of NMRGs in glioma. **(A)** Comparison of the expression levels of the NMRGs in normal brain tissue (GTEx) and glioma (TCGA). **(B)** Comparison of the NMRGs expression in different grades of glioma analyzed in CGGA glioma samples. **(C, D)** GO and KEGG pathway analysis of the NMRGs.

The research workflow diagram for NMRGS construction and corresponding analyses is presented in **Figure 1**. The co-expression relationship among the 40 NMRGs was illustrated in **Supplementary Figure S1**. A comparison of expression levels of 40 NMRGs was made between the TCGA glioma samples and the GTEx normal brain samples. A total of 33 NMRGs were significantly different between the two groups, including 12 downregulated genes and 21 upregulated genes (**Figure 2A**). Subsequently, we further explored the expression levels of NMRGs among different WHO grades. Most NMRGs were significantly correlated with WHO grades analyzed in CGGA glioma samples. (**Figure 2B**). A similar correlation also can be seen in TCGA glioma samples (**Supplementary Figure S2**), suggesting that NMRGs may be involved in the malignant progression of glioma. The Gene Ontology (GO) enrichment and KEGG analyses were performed based on these 40 NMRGs. The top 5 biological processes (BP) and top 5 molecular functions (MF) enriched by GO analysis were presented in **Figure 2C**, indicating that the NMRGs were mainly enriched in projects linked to nicotinamide nucleotide metabolic process, pentosyltransferase activity, ADP-ribosylase activity, nucleotidase activity, and glycosyltransferase activity. The nine enriched pathways are shown in **Figure 2D**, involving Nicotinate and nicotinamide metabolism, biosynthesis of cofactors, pantothenate and CoA biosynthesis, starch and sucrose metabolism, etc.

### NMRGS was constructed based on the expression level and regression coefficient of the six survival-related NMRGs

**Figure 3:**
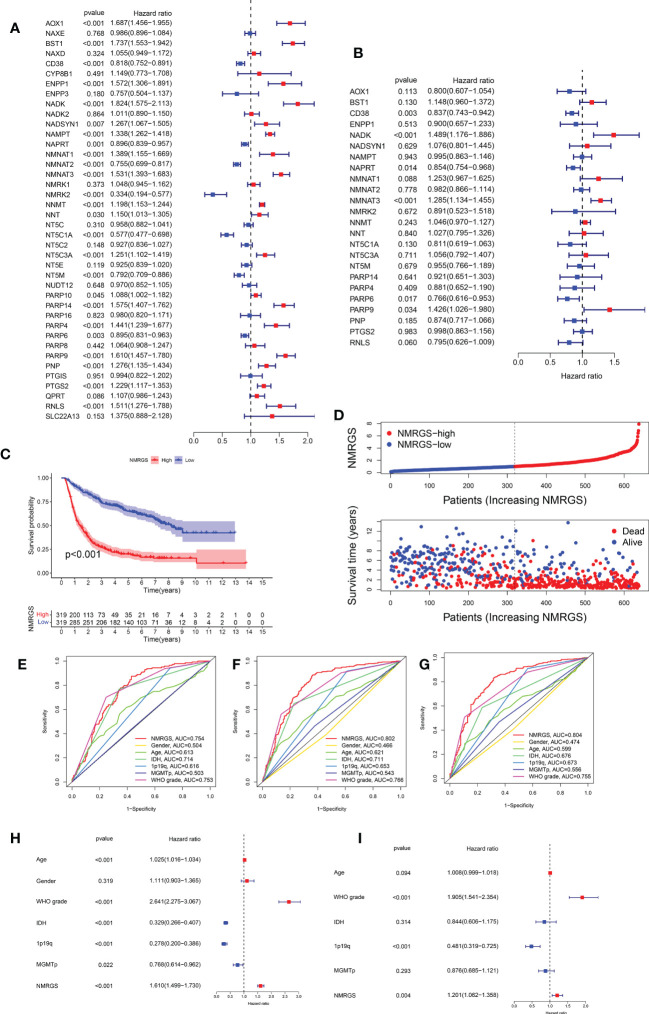
Development and validation of the NMRGS in CGGA693 cohort. **(A)** Univariate Cox regression analysis of the NMRGs. **(B)** Multivariate Cox regression analysis for NMRGS construction. **(C)** Kaplan-Meier analysis for survival in CGGA693 cohort. **(D)** Distribution plot of NMRGS score and survival status of glioma patients in CGGA693 cohort. **(E-G)** ROC curve analyses of NMRGS and the clinicopathological characteristics in predicting 1-, 3-, and 5-year overall survival in CGGA693 cohort. **(H, I)** Univariate and multivariate regression analyses of NMRGS and the clinicopathological characteristics in CGGA693 cohort.

Univariate Cox regression analysis was performed to identify the NMRGs correlated with OS based on the data of the training cohort (**Figure 3A**). Afterward, Kaplan–Meier survival analysis was conducted to filter these genes further. In the end, a total of 24 NMRGs were found associated with prognosis (**Supplementary Figure S3**). Subsequently, these genes were screened out for further multivariate Cox regression analysis. Finally, six NMRGs were found to be independent predictors for OS, including three risky factors (NMNAT3, NADK, PARP9) and three protective factors (CD38, NAPRT, PARP6) (**Figure 3B**). No overfitting within these genes was proved by lasso regression (**Supplementary Figures S4A, B**). NMRGS was constructed based on the multivariate Cox regression coefficient and the expression of six crucial genes. The NMRGS score of every glioma patient was obtained as follows: NMRGS score = (-0.28992*CD38 expression) + (0.38315*NADK expression) + (-0.04654*NAPRT expression) + (0.2211*NMNAT3 expression) + (-0.24486*PARP6 expression) + (0.29991*PARP9 expression).

### NMRGS could accurately predict the prognosis of glioma patients

Taking the median NMRGS score as a cut-off, we divided the glioma patients of the training cohort into NMRGS-low group and NMRGS-high group. The heat map showed the differential clinicopathological characteristics expression levels of six crucial genes in the two NMRGS subgroups (**Supplementary Figure S5**). KM analysis suggested that patients with high NMRGS scores had shorter OS than patients with low NMRGS scores (**Figure 3C**). To further evaluate the accuracy of these six genes in predicting prognosis and OS, individual KMs were added to evaluate the OS for each gene based on TCGA database. In addition, The AUC value of one year (AUC = 0.823), three years (AUC = 0.903), and five years (AUC = 0.821) for ROC analysis revealed that the NMRGS score had a strong ability to predict the survival of patients with glioma, better than individual hub genes (**Supplementary Figure S6**). Consistent trends were observed in different subgroups based on clinicopathological characteristics (**Supplementary Figure S7**). The distribution plot of the NMRGS score and survival status of every glioma patient was displayed in **Figure 3D**. The AUC value of one year (AUC = 0.754), three years (AUC = 0.802), and five years (AUC = 0.804) for ROC analysis revealed that the NMRGS score had a strong ability to predict the survival of patients with glioma, better than other clinicopathological factors (**Figures 3E–G**). Moreover, the univariate and multivariate regression analyses proved that the NMRGS score, 1p19q codeletion status, and WHO grade were independent risk factors for survival in the CGGA693 data cohort (**Figures 3H, I**). The correlation between the NMRGS score and clinicopathological factors was clarified in the training cohort. We also analyzed the correlation between the six NMRGs and clinicopathological factors. The results suggested that the NMRGS score was closely associated with 1p19q codeletion status, isocitrate dehydrogenase (IDH) mutation status, O-6-Methylguanine-DNA Methyltransferase (MGMT) methylation status, and WHO grade (**Supplementary Figures S8**, **S10**). Significant correlation can also be found between the six NMRGs and clinicopathological factors (**Supplementary Figures S11**, **S12**).

**Figure 4:**
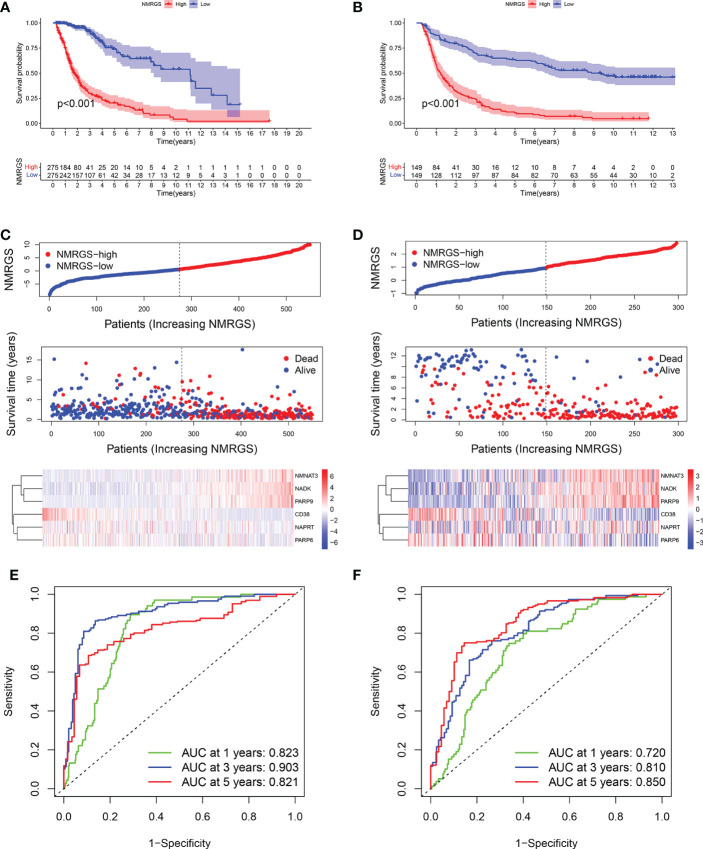
Validation of the NMRGS in TCGA and CGGA325 cohorts. **(A, B)** Kaplan-Meier analysis for survival in TCGA and CGGA325 cohorts. **(C, D)** Distribution plot of NMRGS score and survival status of glioma patients in TCGA and CGGA325 cohorts. **(E, F)** ROC curve analyses of NMRGS in predicting 1-, 3-, and 5-year overall survival in TCGA and CGGA325 cohorts.

Similar analyses were conducted in TCGA and CGGA325 cohorts for validation. Kaplan-Meier curves showed that the OS of patients in the NMRGS-low group was better than that in the NMRGS-high group, indicating that the NMRGS score was a valid prognostic index (**Figures 4A, B**). The NMRGS score and survival status distributions of glioma patients demonstrated that patients in the NMRGS-high group had higher mortality rates than those in the NMRGS-low group (**Figures 4C, D**). The time-dependent ROC analysis revealed that NMRGS score was an OS-predicting index in both TCGA cohort (1-year AUC = 0.823, 3-year AUC = 0.903, 5-year AUC = 0.821) (**Figure 4E**) and CGGA325 cohort (1-year AUC = 0.720, 3-year AUC = 0.810, 5-year AUC = 0.850) (**Figure 4F**). Univariate and multivariate Cox regression analyses revealed that NMRGS score had satisfactory prognostic efficiency independent of clinical factors (**Supplementary Figure S13**). Glioma is molecularly classified into four different subtypes: proneural, neural, mesenchymal, and classical. Further analysis was conducted to assess how these 6 NAD+ metabolism-related genes could benefit the glioma IV subtypes based on TCGA database. Heatmap analysis shows the patient distribution of the IV subtypes (**Supplementary Figure S14A**). Patient fraction of these four different subtypes in NMRGS-low group and NMRGS-high group found significant correlations between NMRGS score and the molecular classification (**Supplementary Figure S14B**). Bar chart statistics further proved this discovery (**Supplementary Figure S14C**). In addition, we analyzed the relationship between the six individual genes and molecular classification. Our results show that all these hub genes, except NAPRT, are associated with molecular classification (**Supplementary Figures S14D–K**). Finally, KM analysis was conducted in different subgroups based on molecular classification. We found that patients with high NMRGS scores had shorter OS than patients with low NMRGS scores in proneural subgroup and neural subgroup but not in mesenchymal subgroup and classical subgroup (**Supplementary Figures S14L–O**). Based on these analyses, we think this NMRGS could benefit the glioma IV subtypes, especially the proneural subgroup and neural subgroup.

### A nomogram was constructed based on NMRGS score, 1p19q codeletion status, and WHO grade

**Figure 5:**
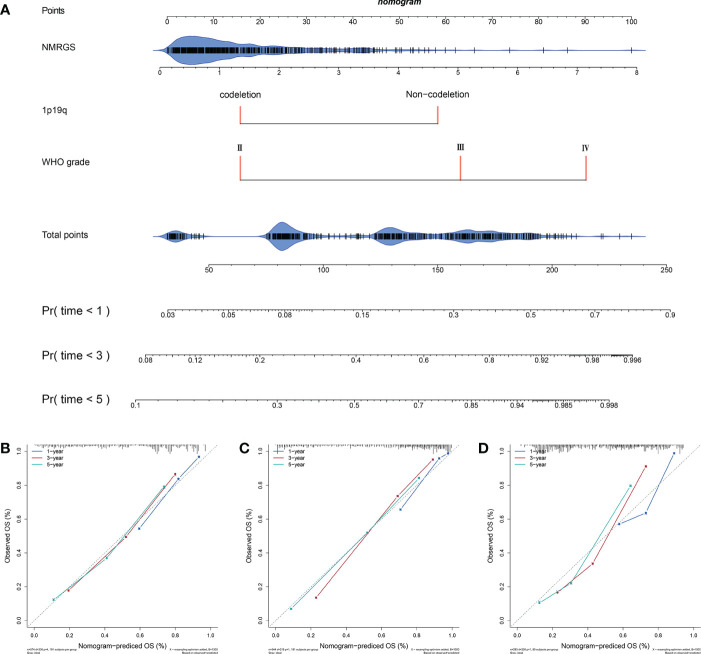
Construction and evaluation of a nomogram. **(A)** A nomogram was constructed based on the independent risk factors (NMRGS score, 1p19q codeletion status, and WHO grade) in CGGA693 cohort. **(B–D)** Calibration curves showing the concordance between the actual and nomogram-predicted 1-, 3-, and 5-year overall survival in CGGA693, TCGA, and CGGA325 cohorts.

A nomogram was constructed based on the independent risk factors (NMRGS score, 1p19q codeletion status, and WHO grade) found in **Figure 3I** to predict the OS of 1, 3, and 5 years, making the NMRGS more applicable for clinical use (**Figure 5A**). Concordance indices (C-indices) were used to evaluate the prediction accuracy of the nomogram, valued at 0.801 ± 0.037 in CGGA693cohort, 0.815 ± 0.037 in TCGA cohort, and 0.749 ± 0.058 in CGGA325 cohort. The calibration plots exhibited a perfect fit between the actual and nomogram-predicted probability in both the training and validation cohorts, revealing that the nomogram had excellent concordance in predicting the OS of 1, 3, and 5 years (**Figures 5B–D**).

### NMRGS score is correlated with immune landscape in glioma

**Figure 6:**
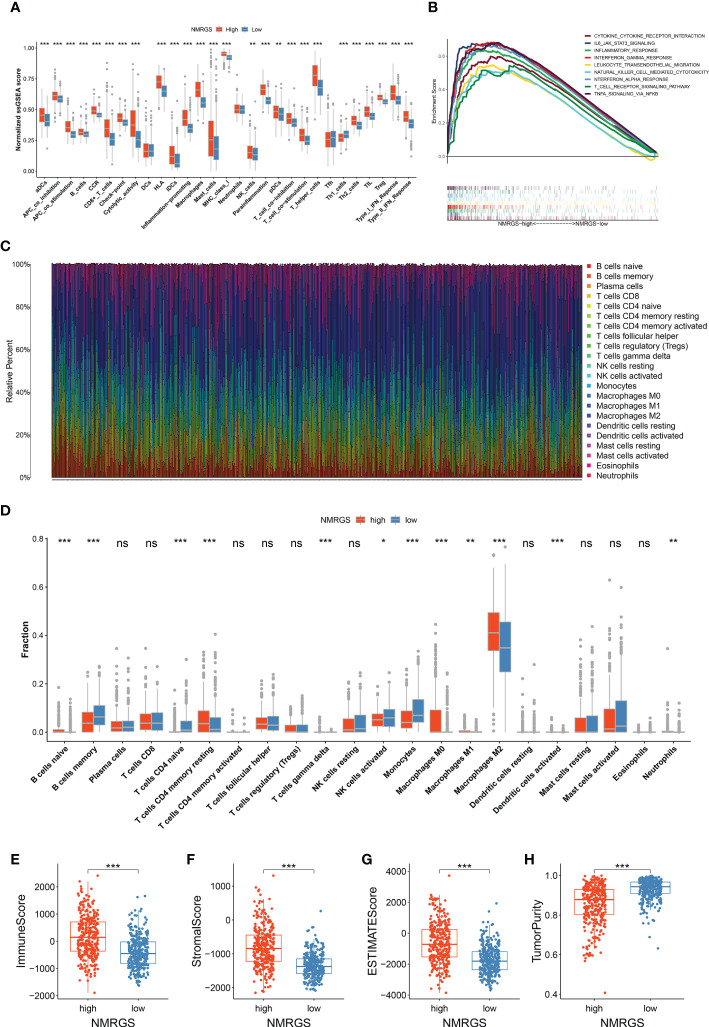
Association between NMRGS and immune landscape in glioma. **(A)** Comparison of ssGSEA scores between NMRGS-low group and NMRGS-high group. **(B)** GSEA analysis of immune-related pathways between NMRGS-low group and NMRGS-high group. **(C)** Proportion of the immune cell infiltration of glioma samples in CGGA693 cohort. **(D)** Comparison of immune cell infiltration between NMRGS-low group and NMRGS-high group. **(E–H)** Comparison of ImmuneScore, StromalScore, ESTIMATEScores, and TumorPurity between NMRGS-low group and NMRGS-high group. ns, not significant; *p < 0.05, **p < 0.01, ***p < 0.001.

Single-sample GSEA analysis showed a remarkable positive correlation between NMRGS score and immune-related functions. Immune cell-related infiltrating scores and immune-related pathways were significantly different between the NMRGS-low group and the NMRGS-high group (**Figure 6A**). Gene set enrichment analysis (GSEA) in the CGGA693 cohort revealed that NMRGS score was associated with immune-related pathways, including IL6-JAK-STAT3-signaling, interferon-gamma response, leukocyte transendothelial migration, natural killer cell-mediated cytotoxicity, t cell receptor signaling pathway and so on (p < 0.05, FDR < 0.25) (**Figure 6B**). In order to comprehensively analyze the immune microenvironment, we used CiberSort to calculate the permeability of 22 immune cells. The immune cell proportion of glioma samples in the CGGA693 cohort was shown in **Figure 6C**. Different immune characteristics were found between the NMRGS-low group and the NMRGS-high group. Higher infiltration of B cells native, CD4+ memory resting T cells, T cells gamma delta, macrophages (M0, M1, and M2), activated dendritic cells, and neutrophils were observed in NMRGS-high group, while B cells memory, CD4+ T cells naive, NK cells activated and monocytes infiltrated more in the NMRGS-low group (**Figure 6D**). In addition, higher ImmuneScore, StromalScore, ESTIMATEScores, and lower TumorPurity were found in the NMRGS-high group. ImmuneScore or StromalScore scores were positively correlated with elevated immunity or stromal ratios, which represented greater proportions of the corresponding components in TME (**Figures 6E–H**). Similar results analyzed on the TCCA and CGGA325 datasets were presented in **Supplementary Figure S15**.

### A robust correlation was found between TMB and NMRGS score

**Figure 7:**
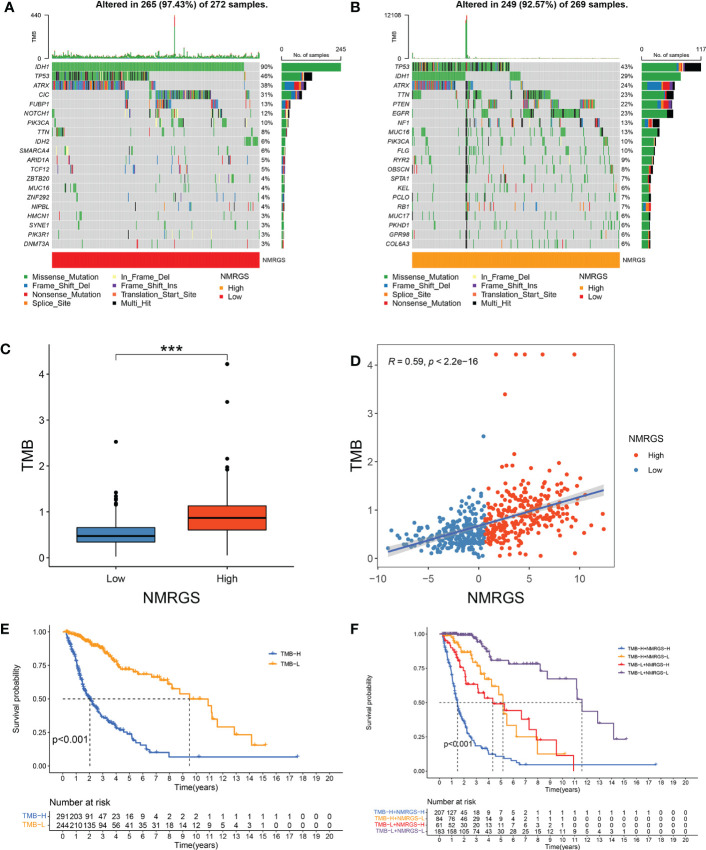
Association between NMRGS score and TMB. **(A)** Top 20 mutated genes in NMRGS-low group. **(B)** Top 20 mutated genes in NMRGS-high group. **(C)** Comparison of TMB between NMRGS-low group and NMRGS-high group. **(D)** The correlation between NMRGS score and TMB. **(E)** KM analysis between the high-TMB group and the low-TMB group. **(F)** Comprehensive KM analysis of the effects of NMRGS and TMB on survival. ***p < 0.001.

TMB was thought to be correlated with the immune infiltration of tumor patients. We analyzed the genetic mutation profile of the TCGA cohort to gain further insight into the immunologic nature of different NRGPI subgroups. In total, somatic mutations were found in 249/269 (92.57%) samples in the NMRGS-high group and 265/272 (97.43%) samples in the NMRGS-low group. The top 20 genes with the highest mutation rates in NMRGS subgroups were identified. **Figures 7A, B** showed different mutation classifications in the NMRGS-low group and the NMRGS-high group. The comparison of TMB between the NMRGS-high group and the NMRGS-low group found that TMB was significantly higher in NMRGS-high patients (**Figure 7C**). In addition, a robust correlation between TMB and NMRGS score was found in **Figure 7D** (R=0.59, p < 2.2e-16). KM analysis was performed to evaluate the influence of the NMRGS score combined with the TMB on survival. Patients in high-TMB group survived shorter than the low-TMB group (**Figure 7E**). More importantly, patients with high NMRGS and high TMB were found have the worst outcome (**Figure 7F**).

### NMRGS score is associated with HLA system

**Figure 8:**
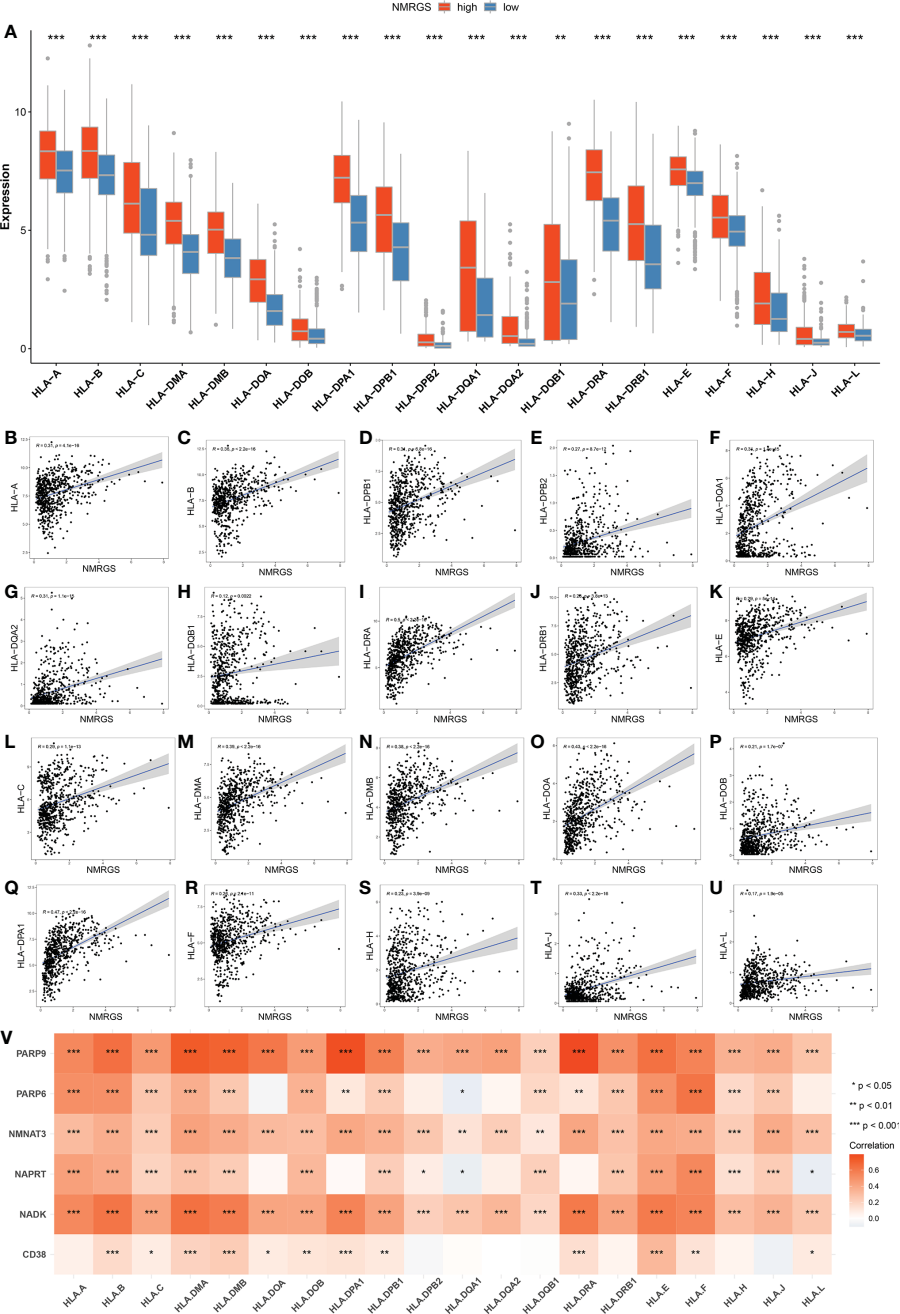
**(A)** Comparison of HLA molecules between NMRGS-low group and NMRGS-high group. **(B–U)** Correlations between HLA molecules and the NMRGS score. **(V)** Correlations between HLA molecules and the six hub NMRGs.

The current understanding of the pathways leading to the restoration of HLA expression could be utilized to conceive immunotherapies. Tumor cells evade immune detection by developing deficiencies in their HLA presentation pathways, allowing important tumor antigens to persist undetected by the immune system. We compared the expression of various HLA molecules between the NMRGS-high group and the NMRGS-low group. The correlation between multiple HLA molecules and NMRGS score has also been further studied. Our study showed that HLA molecules were uniformly under-expressed in the NMRGS-high group compared with the NMRGS-low group (**Figure 8A**; **Supplementary Figures S16A**, **S17A**). Significant positive correlations were found between various HLA molecules and the NMRGS score (**Figures 8B–U**; **Supplementary Figuress S16B–U**, **S17B–U**). In addition, the analysis results show that the six hub NMRGs are also correlated with the HLA system (**Figure 8V**; **Supplementary Figures S16V**, **S17V**).

### NMRGS may have the potential to serve as an indicator for predicting the effectiveness of ICI therapy in glioma

**Figure 9:**
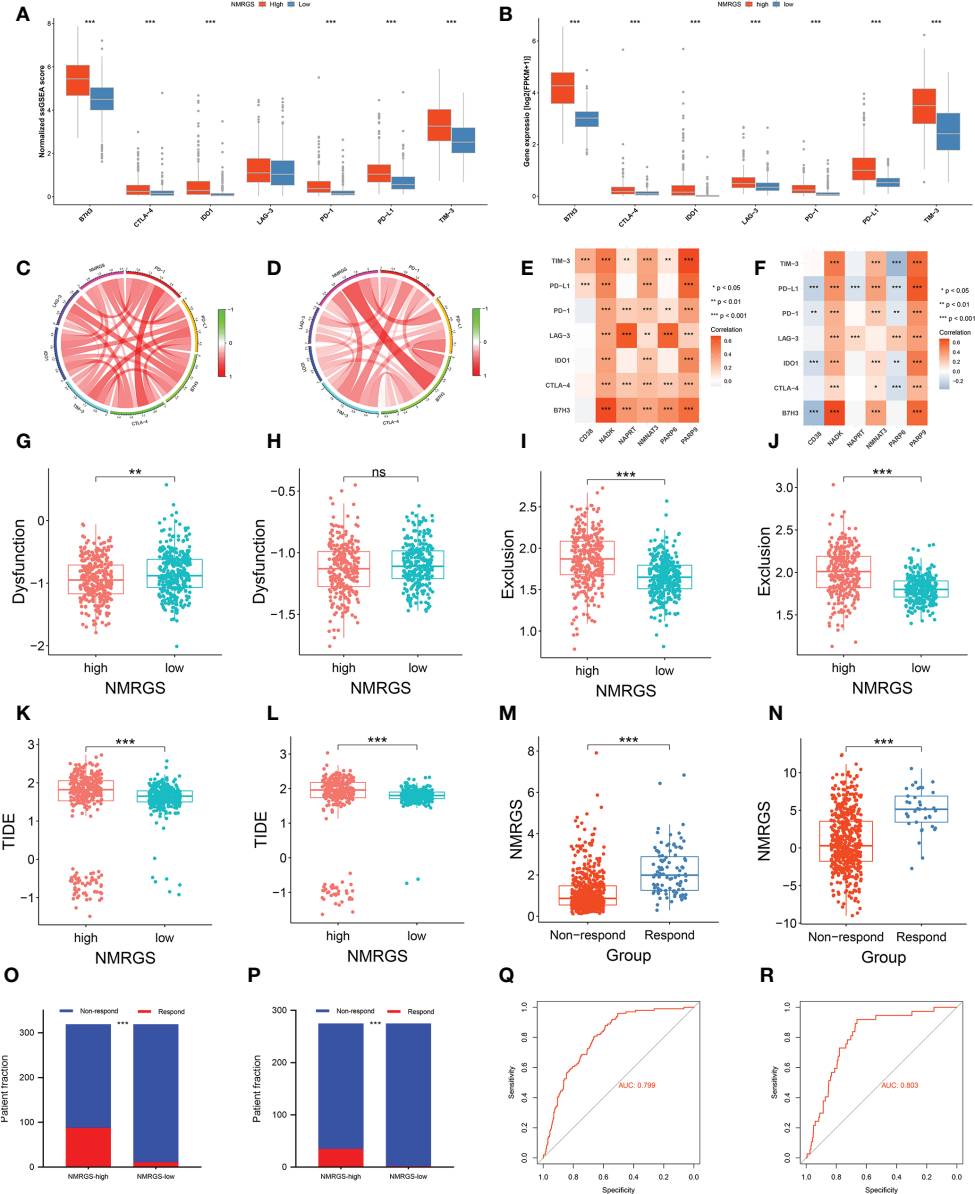
Association between NMRGS and ICI therapy response. **(A, B)** Comparison of representative immune checkpoints between NMRGS-low group and NMRGS-high group in CGGA693 and TCGA cohorts. **(C, D)** Analyses of correlation between NMRGS and representative immune checkpoints expression in CGGA693 and TCGA cohorts. **(E, F)** Analysis of correlation between the hub genes and representative immune checkpoints expression in CGGA693 and TCGA cohorts. **(G–L)** Comparison of dysfunction score, exclusion score, and TIDE score between NMRGS-low group and NMRGS-high group in CGGA693 and TCGA cohorts. **(M, N)** Comparison of NMRGS score between responders and non-responders in CGGA693 and TCGA cohorts. **(O, P)** Patient fraction of responders and non-responders in NMRGS-low group and NMRGS-high group. **(Q, R)** ROC curve analysis of NMRGS in predicting the efficacy of ICI treatment.

The expression level of immune checkpoints is directly related to the therapeutic effect of ICI. Representative immune checkpoints were investigated in both CGGA693, TCGA and CGGA325 cohorts. Significantly higher expression levels were detected in all these represent immune checkpoints between NMRGS-high group and NMRGS-low group (**Figures 9A, B**; **Supplementary Figure S18A**). In addition, we collected 36 other immune checkpoints and compared the expression of these checkpoints between the two groups (**Supplementary Figure S19**). NMRGS score was found to have strong positive correlations with these represent immune checkpoints (**Figures 9C, D**; **Supplementary Figure S18B**). Besides, the correlations between NMRGs and these represent immune checkpoints were investigated (**Figures 9E, F**; **Supplementary Figure S18C**). Further, the tumor immune dysfunction exclusion (TIDE) algorithm was used to evaluate the potential response to ICI therapy.

Lower dysfunction score (**Figures 9G, H**; **Supplementary Figure S18D**), higher exclusion score (**Figures 9I, J**; **Supplementary Figure S18E**), and higher TIDE score (**Figures 9K, L**; **Supplementary Figure S18F**) were found in the NMRGS-high group. In addition, the responders showed to have significantly higher NMRGS scores than that of non-responders (**Figures 9M, N**; **Supplementary Figure S18G**). There were more responders to ICI therapy in NMRGS-high groups compared with NMRGS-low groups in both CGGA693 and TCGA cohorts (CGGA693: 88/319 vs. 11/319, p<0.0001 and TCGA: 35/275 vs. 2/275, p<0.0001; **Figures 9O, P**; **Supplementary Figure S18H**). A high accuracy of NMRGS in predicting ICI response was evident from the ROC curves (AUC of CGGA693 = 0.799, AUC of TCGA = 0.803, AUC of CGGA325 = 0.719; **Figures 9Q, R**; **Supplementary Figure S18I**). In combination, NMRGS may be used as an indicator for predicting the effectiveness of ICI therapy in glioma.

### The six hub NMRGs was differently expressed in glioma tissues compared with adjacent non-tumor tissue samples

**Figure 10:**
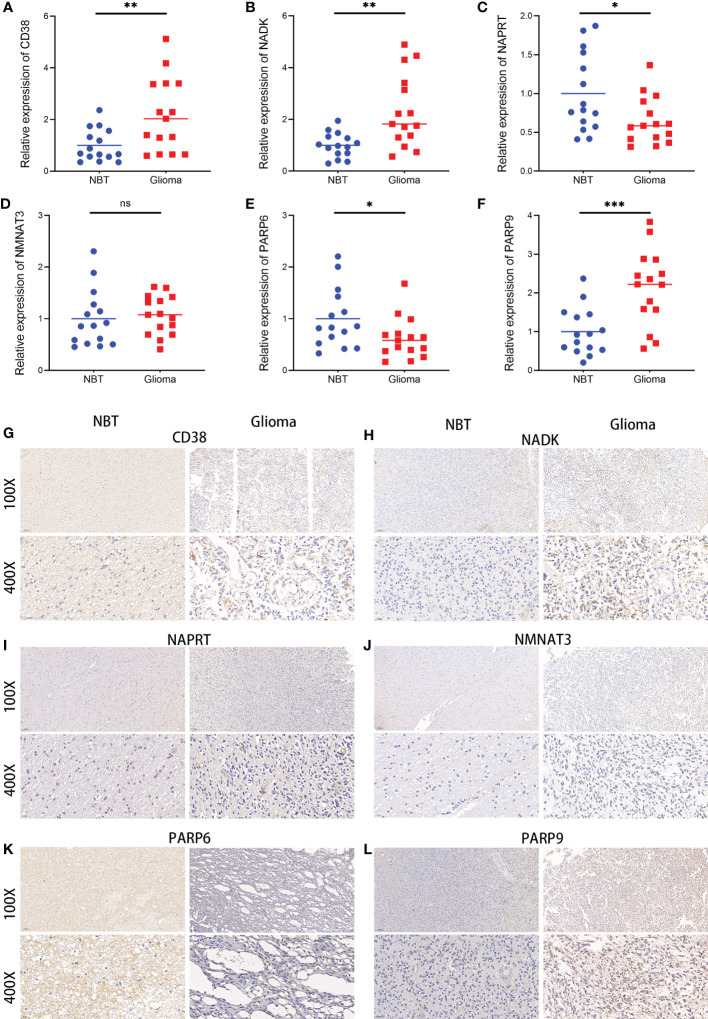
Expressions of the six hub NMRGs in glioma and normal tissues. **(A–F)** The mRNA expressions of the six hub NMRGs in tissues. **(G–L)** Representative IHC staining of the six hub NMRGs in tissues. ns, not significant; *p < 0.05, **p < 0.01, ***p < 0.001.

A validation of the expression patterns of the six hub NMRGs was performed in glioma tissues and adjacent non-tumor tissues, which were seen as normal brain tissues (NBT). According to the results of qRT-PCR, the mRNA expressions of CD38, NADK, PARP9 were significantly elevated in glioma tissues compared with NBT, while the mRNA expressions of NAPRT and PARP6 showed a significant downward trend in glioma tissues (**Figures 10A–F**). Results consistent with the mRNA levels were found through immunohistochemistry staining at the protein level. Representative pictures of IHC staining are shown in **Figures 10G–L**. Besides, western blots of the genes were performed in cell lines, including normal human astroglia (NHA) cell line and seven human glioma cell lines (U251, U87, U118, LN229, T98G, A172, KNS-89). Contrary to expectations, except for NMNAT3, the expression of other hub genes was not consistently overexpressed or underexpressed in these glioma cell lines compared to NHA. Interestingly, the expression of CD38, NADK and NAPRT in these cells showed a similar trend (**Supplementary Figure S20**). Further insight basic research needs to be carried out.

## Discussion

Glioma is the most common primary intracranial tumor. Although progress has been made in diagnosis and treatment, glioma is still cancer with high morbidity and mortality. The application of immunotherapy in glioma holds promise recently. However, the immunotherapeutic effects vary among individuals with glioma, prompting researchers to consider the immunosuppressive tumor microenvironment and the moderate level of immunity in the central nervous system (14). Tumor microenvironments result from changes in tumor cells that support unrestricted growth and proliferation, leading to further alterations in cellular behavior that are critical to tumor progression (15). Different glioma subtypes present different modifications in their microenvironment (16). Cancer cells have increased demands for DNA repair activity and high energetic requirements, as well as a high rate of NAD turnover. It has been shown that NAD+ metabolism impacts a wide range of processes that are dysregulated in cancer. Increasing evidence indicates that NAD+ metabolism is associated with tumor immune microenvironment and response to ICI therapy. It was reported that NAMPT overexpression in glioma cell lines increases tumorigenic properties controlling stem cell pathways and enriching the cancer-initiating cell population (17). NAD+ metabolism was found to maintain inducible PD-L1 expression to drive tumor immune evasion (11). NAD+ depletion was found to radiosensitize 2-DG-treated glioma cells by abolishing metabolic adaptation (18). These previous findings have aroused our interest in systematically exploring the prognostic value of these NMRGs and their relationship with anti-tumor immunity.

In this study, 40 NMRGs were identified, 6 of which were selected to construct the NMRGS. No matter for the training cohort (CGGA693) or the validation cohorts (TCGA and CGGA325), the NMRGS showed robust capacity in predicting the survival outcomes of glioma patients, which is demonstrated by survival analysis, ROC curve, and Cox regression analysis. A nomogram with improved predictive capacity was developed by combining the prognostic NMRGS with other independent prognostic factors (1p19q codeletion status and WHO grade). Biological processes and pathways associated with immunity were identified in functional enrichment analyses. We further uncovered the differential immune landscape between the two risk subgroups by comparing the abundance of immune cells, immune and stromal scores, and expression levels of immunoregulatory molecules. Additionally, the NMRGS score may be associated with differences in ICI therapy efficacy.

The NMRGS comprised six crucial genes, including CD38, NADK, NAPRT, NMNAT3, PARP6, and PARP9. The first key gene, CD38, is the major NAD-hydrolyzing ectoenzyme in most mammals and has recently been implicated in regulating metabolism and the pathogenesis of cancers (19). However, the understanding of the impact of CD38 on tumor progression remains limited, ambiguous, and controversial. CD38 was found to function as a tumor‐promoting factor in melanoma, esophageal, and lung cancers, though conflicting data does exist on the influence of CD38 in the progression of prostate cancer (20). CD38 expression on tumor cells was reported to rise in murine and human origins in response to PD-L1 antibody therapy, which led to dysfunction of tumor-infiltrating CD8 T cells due to increasing adenosine production (21). The second key gene, NADK, uses ATP as a phosphate donor to phosphorylate NAD+ to NADP, which is then reduced to nicotinamide adenine dinucleotide phosphate (NADPH). NADPH plays an essential role in proliferating cancer cells and neutralizing the dangerously high levels of reactive oxygen species produced by metabolic activity (22–24). Given its role in metabolism and ROS regulation, it is unsurprising that several recent studies have identified NADK as a potential therapeutic target for cancer treatment (25, 26). The third key gene, NAPRT, is responsible for the first step in converting nicotinic acid to NAD. The primary NAD salvageable precursor in human cells is nicotinamide, catalyzed by NAMPT. Nicotinic acid is more effective at increasing NAD levels, so some tissues may preferentially use the nicotinic acid salvage pathway (27). By causing metabolic stress, inhibition of NAMPT offers a novel therapeutic approach in tumors that lack NAPRT expressions, such as glioblastoma and lymphoma. At the same time, normal cells are saved by NA by activating the NAPRT pathway (28–30). The fourth key gene, NMNAT3, is a member of the NMNAT family. NMNAT is another rate-limiting enzyme reversibly catalyzing the critical step in the biosynthesis of NAD from ATP and NMN (31). NMNAT was found to enhance NAD-dependent posttranslational modifications of p53 and deacetylation of p53 by inhibiting DNA damage-p53-caspase-3 signaling pathway, allowing glial cells with harmful mutations to survive and multiply (32). Besides, A study found that depletion of NMNAT-2 led to increased polysome association, enhanced translation of specific mRNAs, and decreased ovarian cancer growth (33). The remaining two key genes, PARP6 and PARP9, are all members of Poly(ADP-ribose) polymerase (PARP) family and are involved in the genesis and development of some tumors (34). PARP6 was confirmed can inhibit the expression of XRCC6 by inducing degradation and thus regulate the Wnt/β-catenin pathway, which contributes to the suppression of hepatocellular carcinoma (35). However, pharmacological inhibition of PARP6 triggered multipolar spindle formation and led to apoptosis in a subset of breast cancer cells and antitumor effects (36). As for PARP9, it has a carboxy-terminal amino acid sequence similar to other PARPs but lacks PARP activity. The overexpression of PARP9 has been demonstrated to positively correlate with the pathological progression of lymphoma, breast cancer, and prostate cancer (37–40). In summary, these six selected NMRGs were directly or indirectly involved in regulating NAD+ Metabolism in cancer.

Recent discoveries have revealed that the glioma microenvironment affects tumorigenesis and its response to immunotherapeutic treatment. A deep understanding of the complex heterogeneity of glioma immune microenvironment may be the key to unleashing the full potential of immunotherapy strategies. In our study, we found significant differences in TME among NMRGS subgroups, especially in the tumor immune microenvironment. GSEA and ssGSEA showed different immune functional statuses between the two groups, including Interferon response, NK cell-mediated cytotoxicity, cytolytic activity, MHC class I expression, and so on. Glioma cells deprived nutrients and secreted interferential metabolites to remold the immune microenvironment, which activated anti-inflammatory and tolerant mechanisms and hindered anti-tumor responses. Metabolic remodeling injured the tumor recognition, phagocytosis and lysis of cytotoxic T lymphocytes, natural killer cells, glioma-associated macrophages, and dendritic cells, conferred immune silencing phenotypes on dendritic cells and macrophages, and promoted the expansion and infiltration of immunosuppressive regulatory T cells and myeloid-derived suppressor cells. Tumor-associated macrophages recruited to the glioma microenvironment can release growth factors and cytokines in response to the activity of cancer cells, exhibiting an immunosuppressive M2 phenotype and functional behavior (41). Our findings showed that M2-type macrophages were overwhelmingly numerically superior to other immune cells, which is consistent with previous research in the scientific community. In the NMRGS high group with poor survival outcomes, we found significantly higher enrichment of M2 macrophages, indicating that the NMRGS high group had higher immunosuppressive characteristics. Natural killer cells are at the forefront of the body’s defense system and are powerful effectors of the anticancer immune response, rapidly recognizing and killing tumor cells with little response to healthy tissue. Evidence has shown that the infiltration and cytotoxicity of NK cells in cancer tissues affect therapeutic efficacy and survival, but their function is often influenced by factors released by tumors or other immunosuppressive cells (42). In our study, the infiltration of activated NK cells in NMRGS-high group was less than that in NMRGS-low group, which further reflected that NMRGS-high group had a higher state of immunosuppression. Our results suggest that the interaction between glioma cell NAD+ metabolism and immune cells in the microenvironment may provide a new perspective for understanding glioma immune escape and immunotherapy refractory.

Inhibitory and costimulatory receptors, known as “immune checkpoints,” are one of the mechanisms by which cancer cells evade immune surveillance, induce immune tolerance and escape immune destruction (43). In recent years, immune checkpoint inhibitors have revolutionized treatment options for many cancers. These treatments have demonstrated higher efficacy and less toxicity than standard cytotoxic chemotherapy, with some patients experiencing lasting long-term disease control and even remission (44). However, the therapeutic effect of ICI varies significantly among patients. Given this variability, it is increasingly important to identify appropriate biomarkers to select patients for ICI treatment (45). The main intrinsic driver of tumor heterogeneity is genomic alterations. TMB, traditionally defined as the number of non-synonymous exon mutations per megabase (Mut/Mb), has recently been identified as a promising new biomarker for the therapeutic response of ICI (46). A large number of mutations in the exon region of somatic cells will lead to the increase of neoantigen and increase the immunogenicity of cells, thus improving the immune response. Based on this hypothesis, individuals with higher TMB tend to have stronger immune responses. In our study, we observed a strong relationship between NMRGS and TMB, and the correlation coefficient reached 0.59. The group with high NMRGS showed higher TMB than those with low NMRGS, suggesting that the NMRGS-high group may respond better to ICI treatment. The expression of immune checkpoint is the most direct biomarker of ICI treatment (47). Ligands expressed by glioma cells recognize and bind chaperone proteins (receptors) on the surface of immune cells, which play an important role in maintaining peripheral immune tolerance and controlling inflammatory overreaction (48). The results showed that the expression of inhibitory immune checkpoints such as PD-L1 was higher in the NMRGS-high group, further indicating that ICI treatment may be more effective in the NMRGS-high group. By calculating the genome-wide expression profile of patients before treatment and simulating tumor immune escape of different levels of cytotoxic T lymphocytes, Peng Jiang et al. established an online TIDE score combining the characteristics of T cell dysfunction and T cell exclusion to predict the response to immunotherapy (49). In prospective clinical trials, TIDE score has shown outstanding superiority over other biomarkers in predicting ICI response to treatment, and has been recognized as an effective method for predicting immunotherapy response in solid tumor patients (50). Our results showed that TIDE and dysfunction scores were lower in the high NMRGS group, while exclusion scores showed an opposite trend. A higher proportion of patients in the NMRGS-high group responded to ICI therapy than in the NMRGS-low group. Analysis of the ROC curve confirmed that NMRGS is an excellent predictor of ICI response. All in all, our results suggest that NMRGS may be used as a potential ICI therapy management indicator.

In this study, we developed an NMRGS to predict glioma prognosis and ICI treatment. However, it is worth noting that there are still certain limitations in the present study. Firstly, further experiments are needed to elucidate the roles and specific mechanisms of the six hub genes in glioma. Secondly, retrospective data were used to construct and validate NMRGS. Multi-center prospective studies are needed to validate its clinical value and make it more convincing. Finally, we indirectly assessed the potential of NMRGS immunotherapeutic response with TIDE, more studies are needed to confirm this conclusion.

## Data availability statement

Publicly available datasets were analyzed in this study. This data can be found here: CGGA (http://www.cgga.org.cn/) and TCGA (https://portal.gdc.cancer.gov/) websites.

## Ethics statement

The studies involving human participants were reviewed and approved by Union Hospital, Tongji Medical College, Huazhong University of Science and Technology. The patients/participants provided their written informed consent to participate in this study.

## Author contributions

CJ, YZ, and LY conducted this study. JZ drew the figures and tables. XJ, JL, and XW made some revisions of the manuscript. All authors contributed to the article and approved the submitted version.

## Supplementary material

Supplementary Figure 1 Correlation analysis between the 40 NMRGs.

Supplementary Figure 2 Comparison of the NMRGs expression in different grades of glioma NMRGs analyzed in TCGA glioma samples.

Supplementary Figure 3 Kaplan–Meier curve analyses of the NAD+ metabolism-related genes.

Supplementary Figure 4 Lasso regression of the six hub genes. **(A)** Tuning parameter(λ) in LASSO model using cross-validation. **(B)** The LASSO coefficient profile of 6 NMRGs.

Supplementary Figure 5 Distribution of clinicopathological characteristics and expression levels of the hub NMRGs in NMRGS-high group and NMRGS-low group.

Supplementary Figure 6 Further evaluation of the accuracy of these six genes in predicting prognosis and OS based on TCGA cohorts. **(A–F)** Individual KMs of the hub genes. **(G–I)** ROC curve analyses of NMRGS and the six genes in predicting 1-, 3-, and 5-year overall survival in TCGA cohorts.

Supplementary Figure 7 KM analysis in different subgroups based on clinicopathological characteristics.

Supplementary Figure 8 Patients fraction in different clinicopathological subgroups in CGGA693 cohort.

Supplementary Figure 9 Patients fraction in different clinicopathological subgroups in TCGA cohort.

Supplementary Figure 10 Patients fraction in different clinicopathological subgroups in CGGA325 cohort.

Supplementary Figure 11 Comparison of CD38 **(A)**, NADK **(B)**, NAPRT **(C)** expression among different clinicopathological subgroups in CGGA693 cohort.

Supplementary Figure 12 Comparison of NMNAT3 **(A)**, PARP6 **(B)**, PARP9 **(C)** expression among different clinicopathological subgroups in CGGA693 cohort.

Supplementary Figure 13
**(A, B)** Univariate and multivariate Cox regression analyses were conducted in TCGA cohort. **(C, D)** Univariate and multivariate Cox regression analyses conducted in CGGA325 cohort.

Supplementary Figure 14 The correlation between NMRGS and the four molecular subtypes of glioma. **(A)** Distribution of the IV subtypes in NMRGS-low group and NMRGS-high group. **(B)** Patient fraction of these four different subtypes in NMRGS-low group and NMRGS-high group. **(C–K)** the relationship between the six individual genes and molecular classification. **(L–O)** KM analysis in different subgroups based on molecular classification. (PN, proneural subgroup; NE, neural subgroup; ME, mesenchymal subgroup; CL, classical subgroup).

Supplementary Figure 15
**(A, B)** Comparison of immune cell infiltration between NMRGS-low group and NMRGS-high group in TCGA and CGGA325 cohorts. **(C–F)** Comparison of ImmuneScore, StromalScore, ESTIMATEScores and TumorPurity between NMRGS-low group and NMRGS-high group in TCGA cohort. **(H–K)** Comparison of ImmuneScore, StromalScore, ESTIMATEScores and TumorPurity between NMRGS-low group and NMRGS-high group in CGGA325 cohort.

Supplementary Figure 16 Association between NMRGS score and HLA molecules analyzed in TCGA cohort **(A)** Comparison of HLA molecules between NMRGS-low group and NMRGS-high group. **(B–U)** Correlations between HLA molecules and the NMRGS score. **(V)** Correlations between HLA molecules and the six hub NMRGs.

Supplementary Figure 17 Association between NMRGS score and HLA molecules analyzed in CGGA325 cohort **(A)** Comparison of HLA molecules between NMRGS-low group and NMRGS-high group. **(B–U)** Correlations between HLA molecules and the NMRGS score. **(V)** Correlations between HLA molecules and the six hub NMRGs.

Supplementary Figure 18 Association between NMRGS score and ICI therapy response analyzed in CGGA325 cohort. **(A)** Comparison of representative immune checkpoints between NMRGS-low group and NMRGS-high group in CGGA325 cohort. **(A)** Analysis of correlation between NMRGS and representative immune checkpoints expression in CGGA325 cohort. **(A)** Analysis of correlation between the hub genes and representative immune checkpoints expression in CGGA325 cohort. **(D–F)** Comparison of dysfunction score, exclusion score and TIDE score between NMRGS-low group and NMRGS-high group in CGGA325 cohort. **(G)** Comparison of NMRGS score between responders and non-responders in CGGA325 cohort. **(H)** Patient fraction of responders and non-responders in NMRGS-low group and NMRGS-high group. **(I)** ROC curve analysis of NMRGS in predicting the efficacy of ICI treatment analyzed in CGGA325 cohort.

Supplementary Figure 19 Comparison of other immune checkpoints expression between NMRGS-low group and NMRGS-high group in CGGA693, TCGA, and CGGA325 cohorts.

Supplementary Figure 20 Western blots of the hub genes in cell lines.
