# Supplementary figures and images for: A prognostic NAD+ metabolism-related gene signature for predicting response to immune checkpoint inhibitor in glioma
Source: Front Oncol. 2023 Feb 8;13:1051641. doi: 10.3389/fonc.2023.1051641 (PMC9945104; doi:10.3389/fonc.2023.1051641)

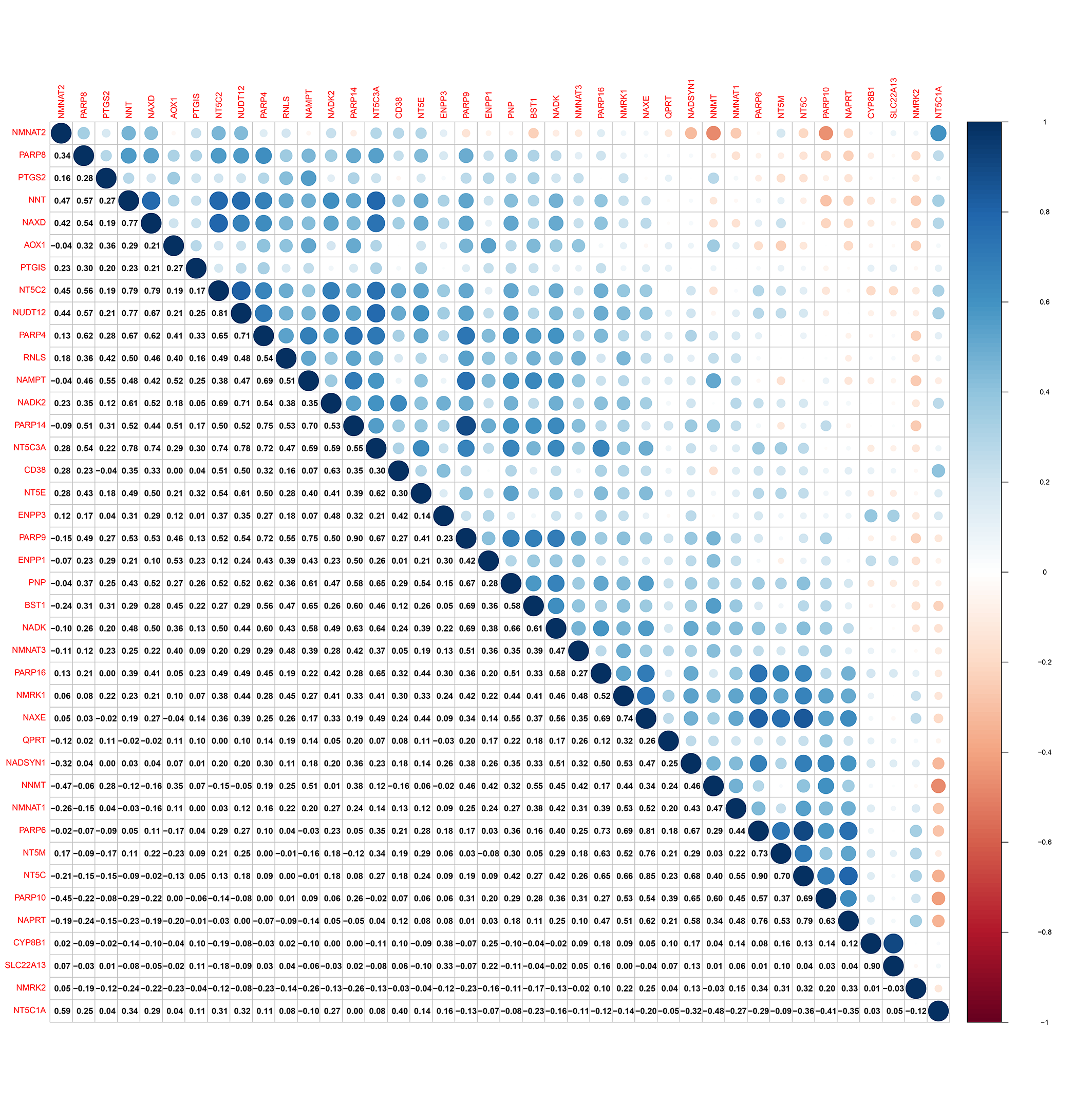

Supplement: Supplementary Figure 1 — Correlation analysis between the 40 NMRGs. [file Image_1.tif]

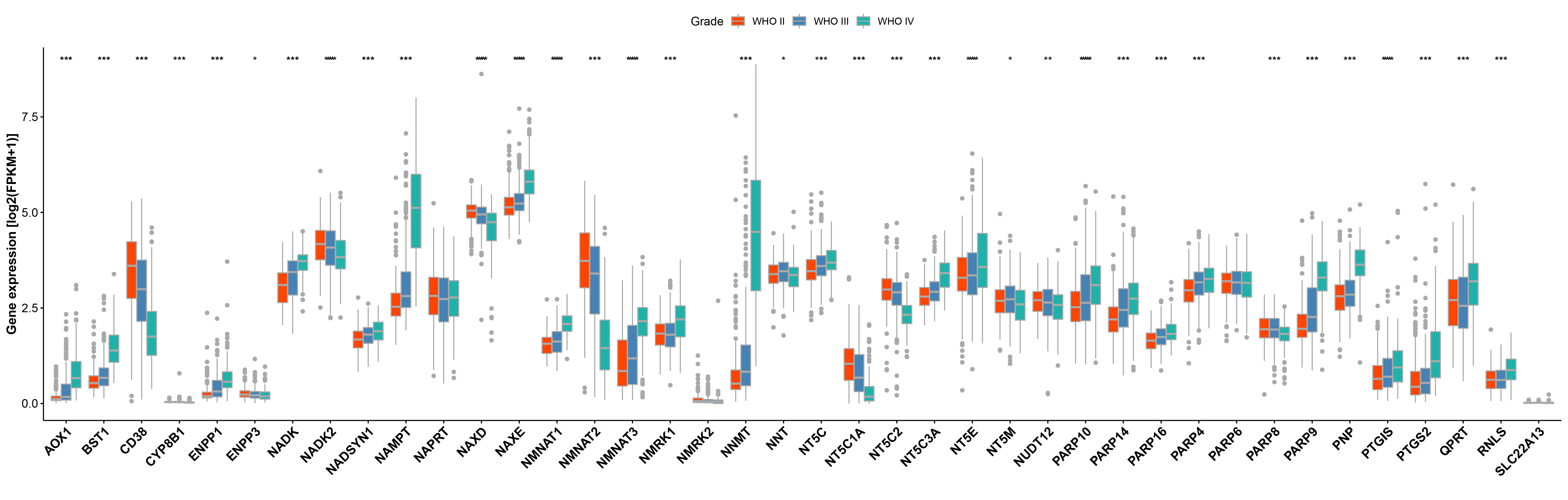

Supplement: Supplementary Figure 2 — Comparison of the NMRGs expression in different grades of glioma NMRGs analyzed in TCGA glioma samples. [file Image_2.tif]

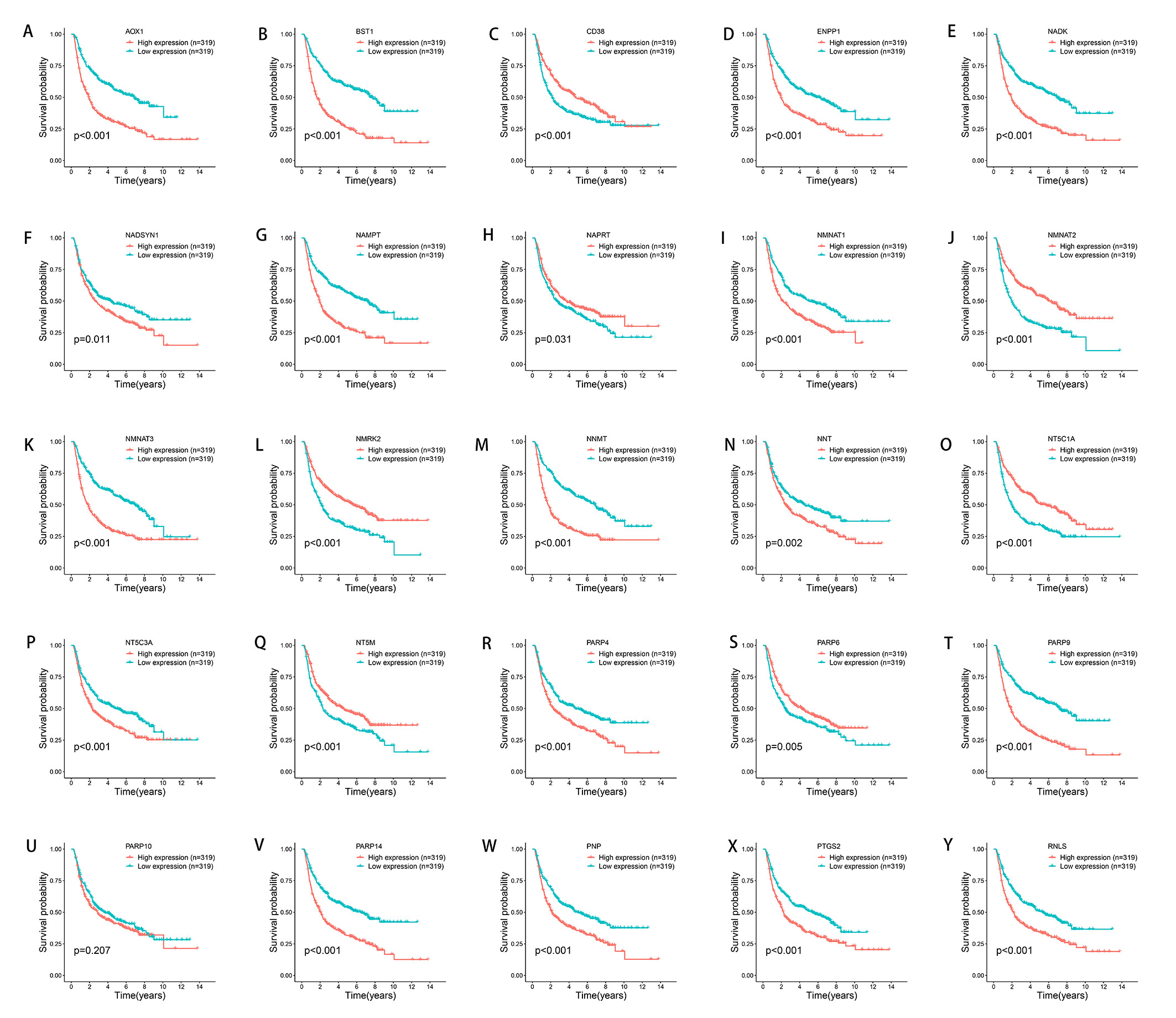

Supplement: Supplementary Figure 3 — Kaplan–Meier curve analyses of the NAD+ metabolism-related genes. [file Image_3.tif]

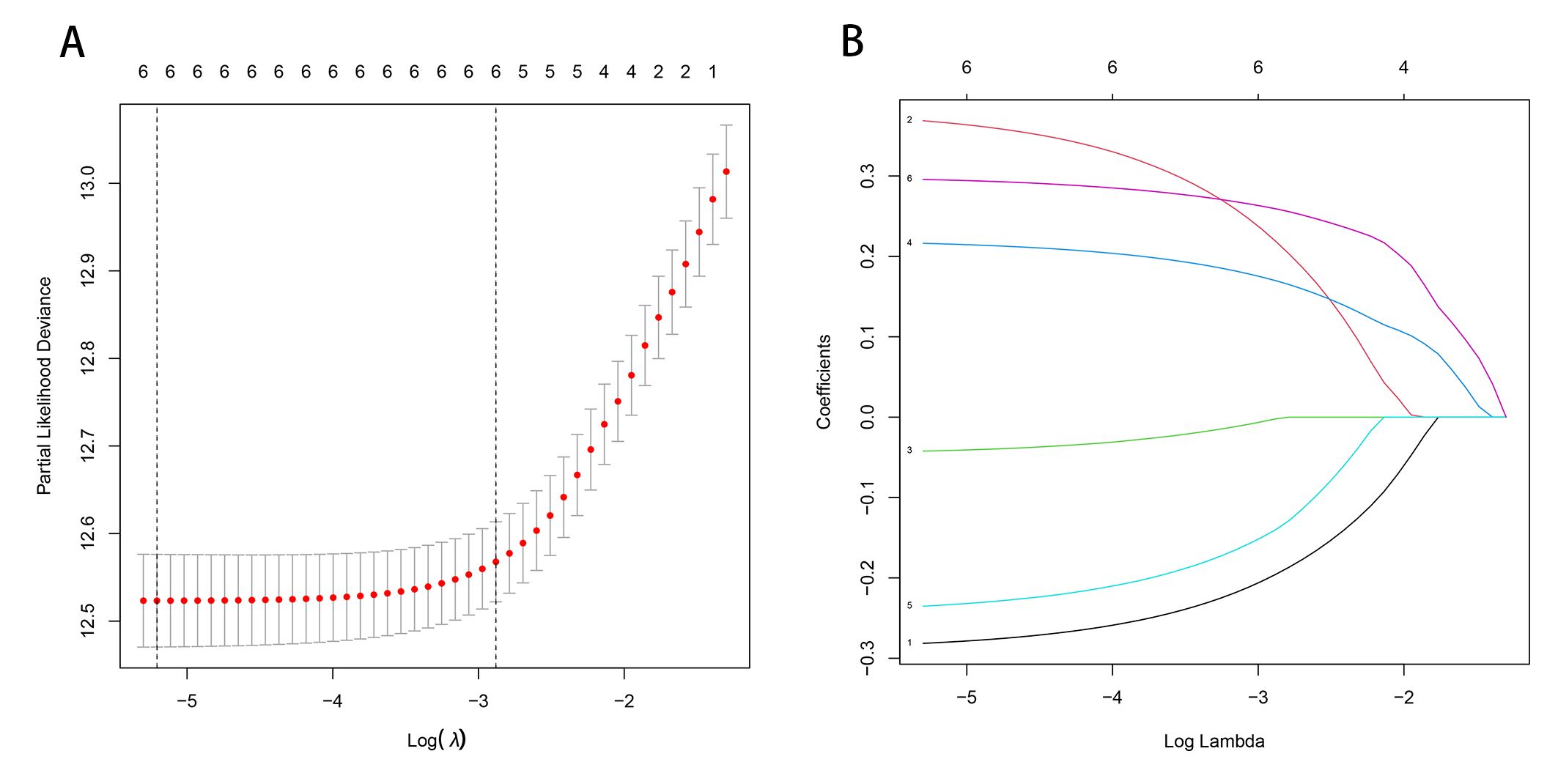

Supplement: Supplementary Figure 4 — Lasso regression of the six hub genes. (A) Tuning parameter(λ) in LASSO model using cross-validation. (B) The LASSO coefficient profile of 6 NMRGs. [file Image_4.tif]

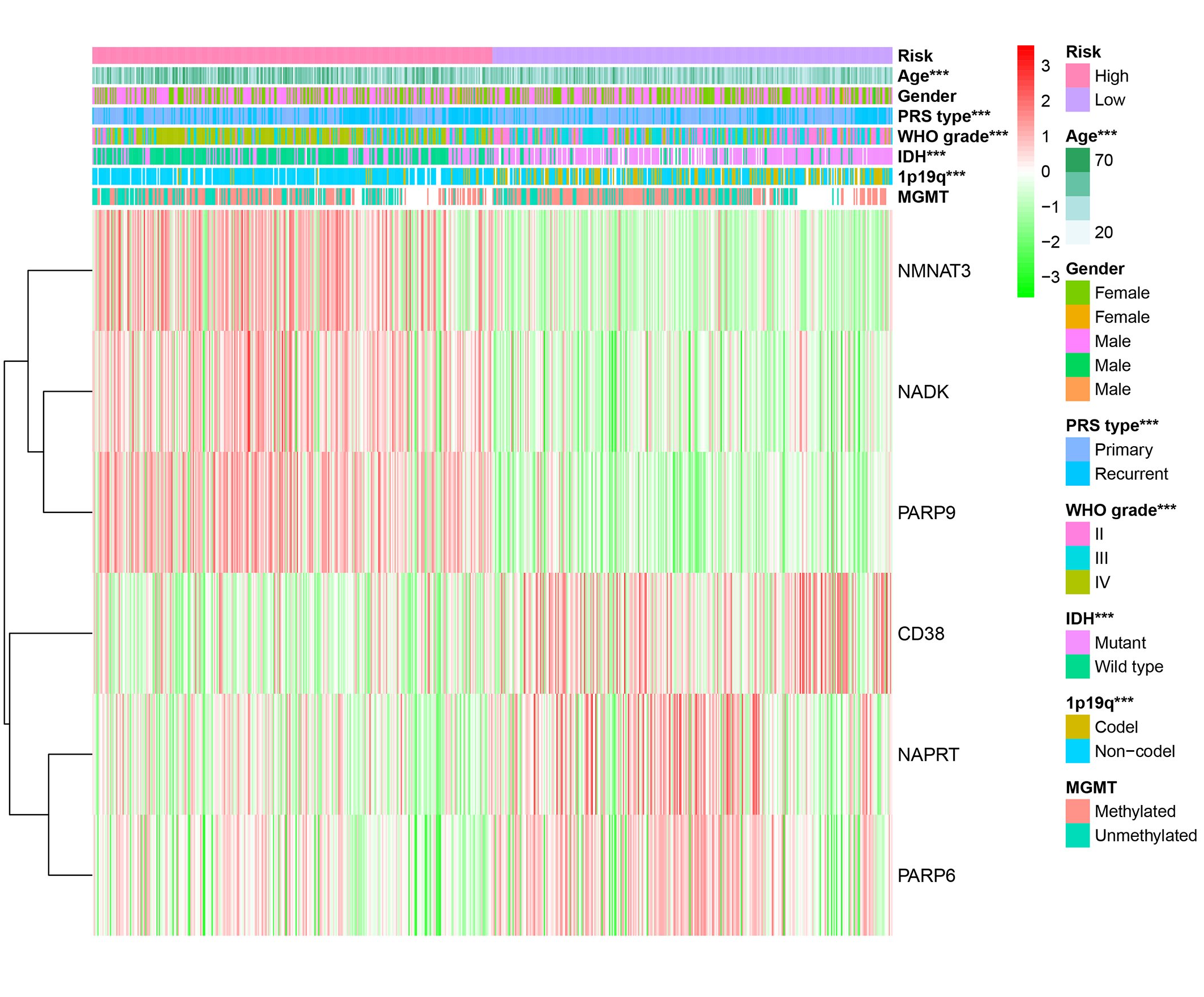

Supplement: Supplementary Figure 5 — Distribution of clinicopathological characteristics and expression levels of the hub NMRGs in NMRGS-high group and NMRGS-low group. [file Image_5.tif]

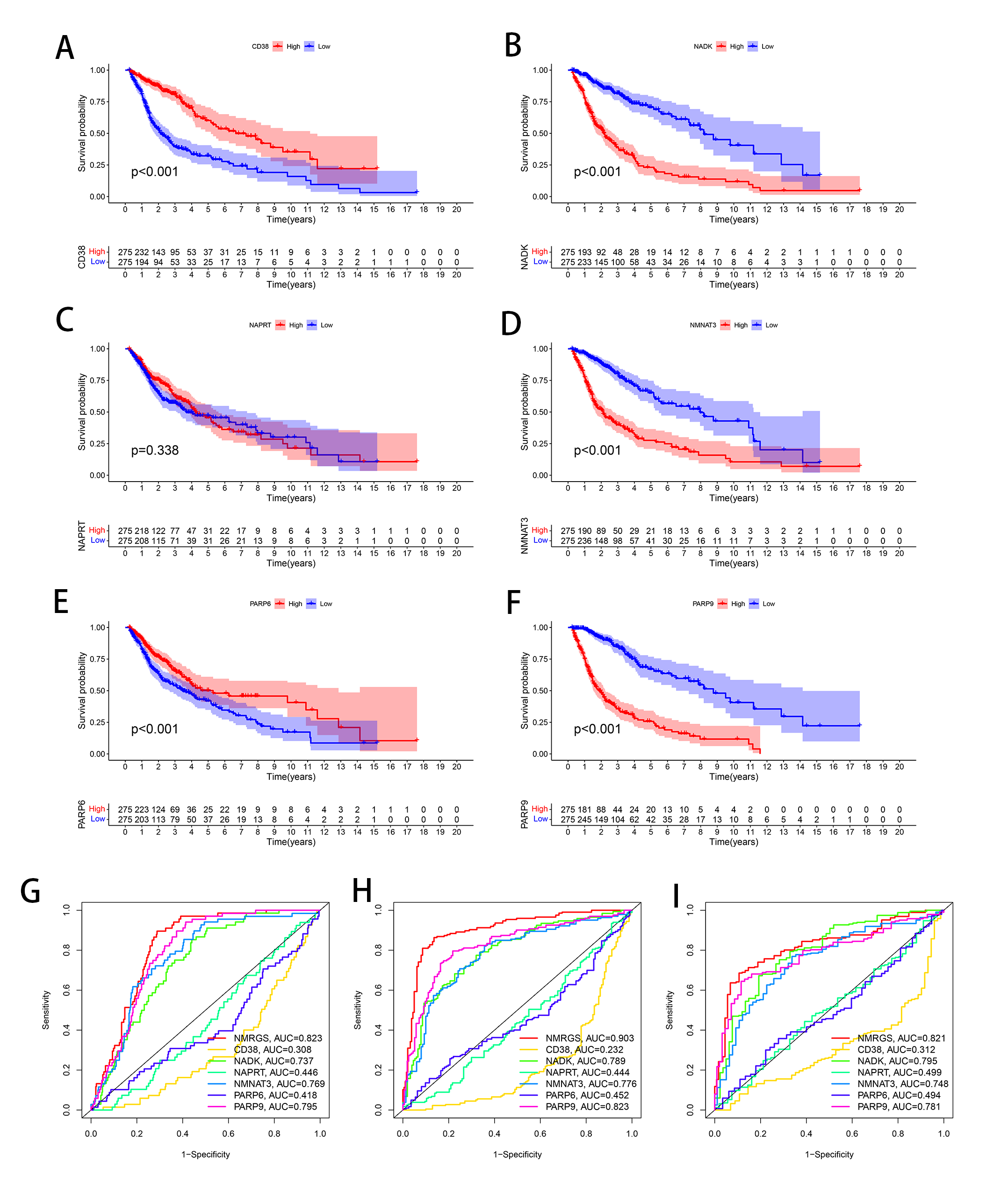

Supplement: Supplementary Figure 6 — Further evaluation of the accuracy of these six genes in predicting prognosis and OS based on TCGA cohorts. (A–F) Individual KMs of the hub genes. (G–I) ROC curve analyses of NMRGS and the six genes in predicting 1-, 3-, and 5-year overall survival in TCGA cohorts. [file Image_6.tif]

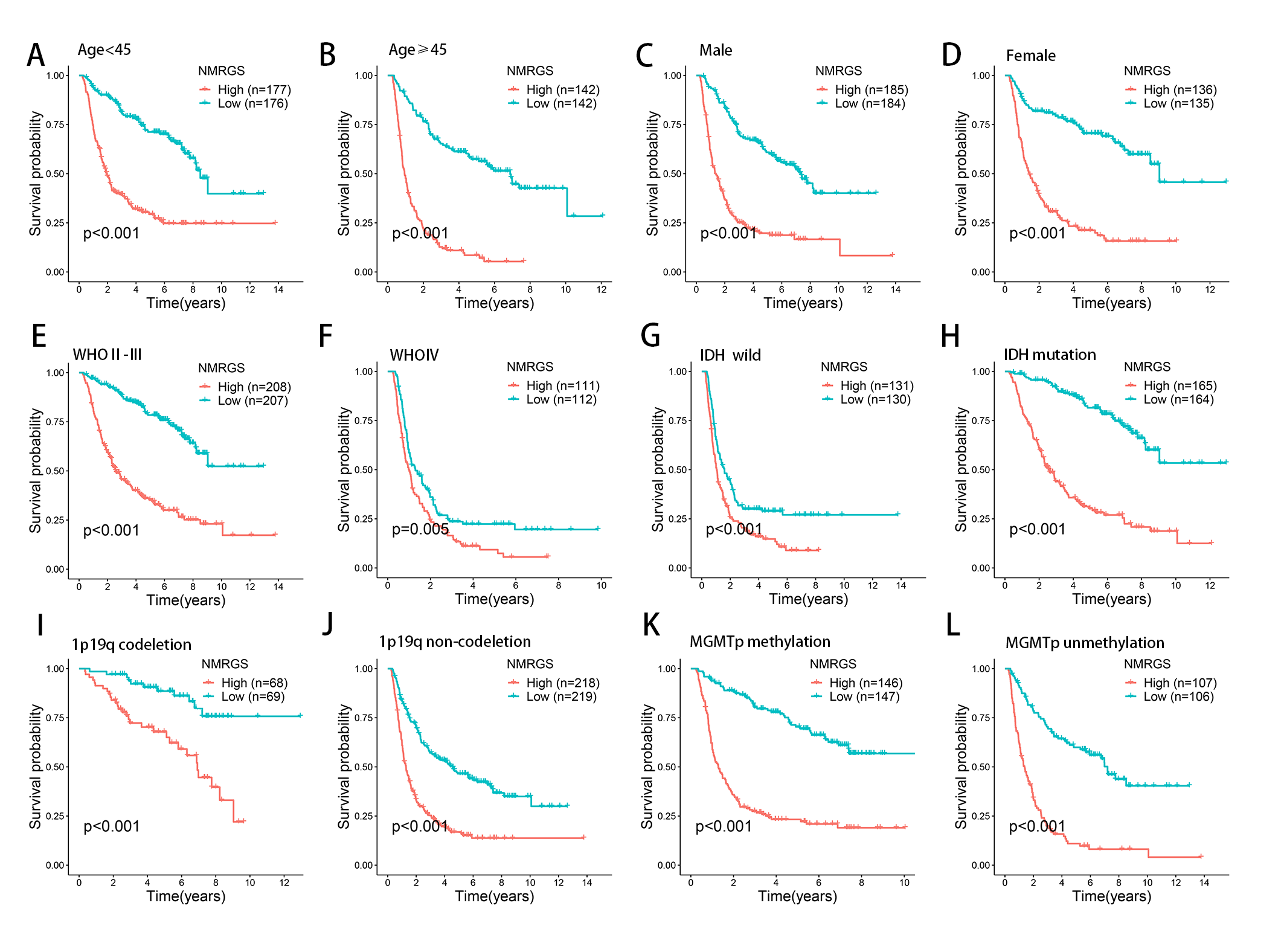

Supplement: Supplementary Figure 7 — KM analysis in different subgroups based on clinicopathological characteristics. [file Image_7.tif]

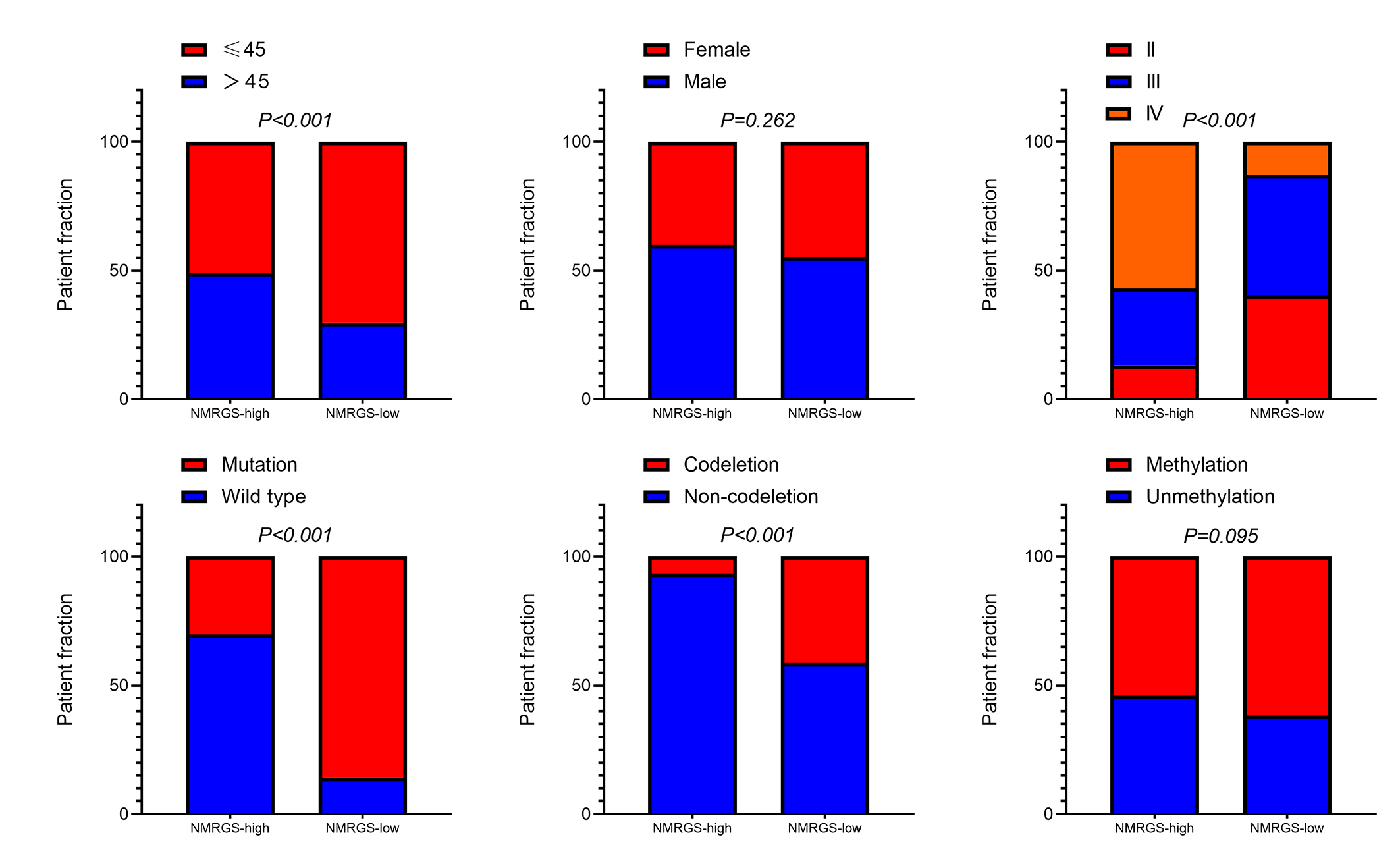

Supplement: Supplementary Figure 8 — Patients fraction in different clinicopathological subgroups in CGGA693 cohort. [file Image_8.tif]

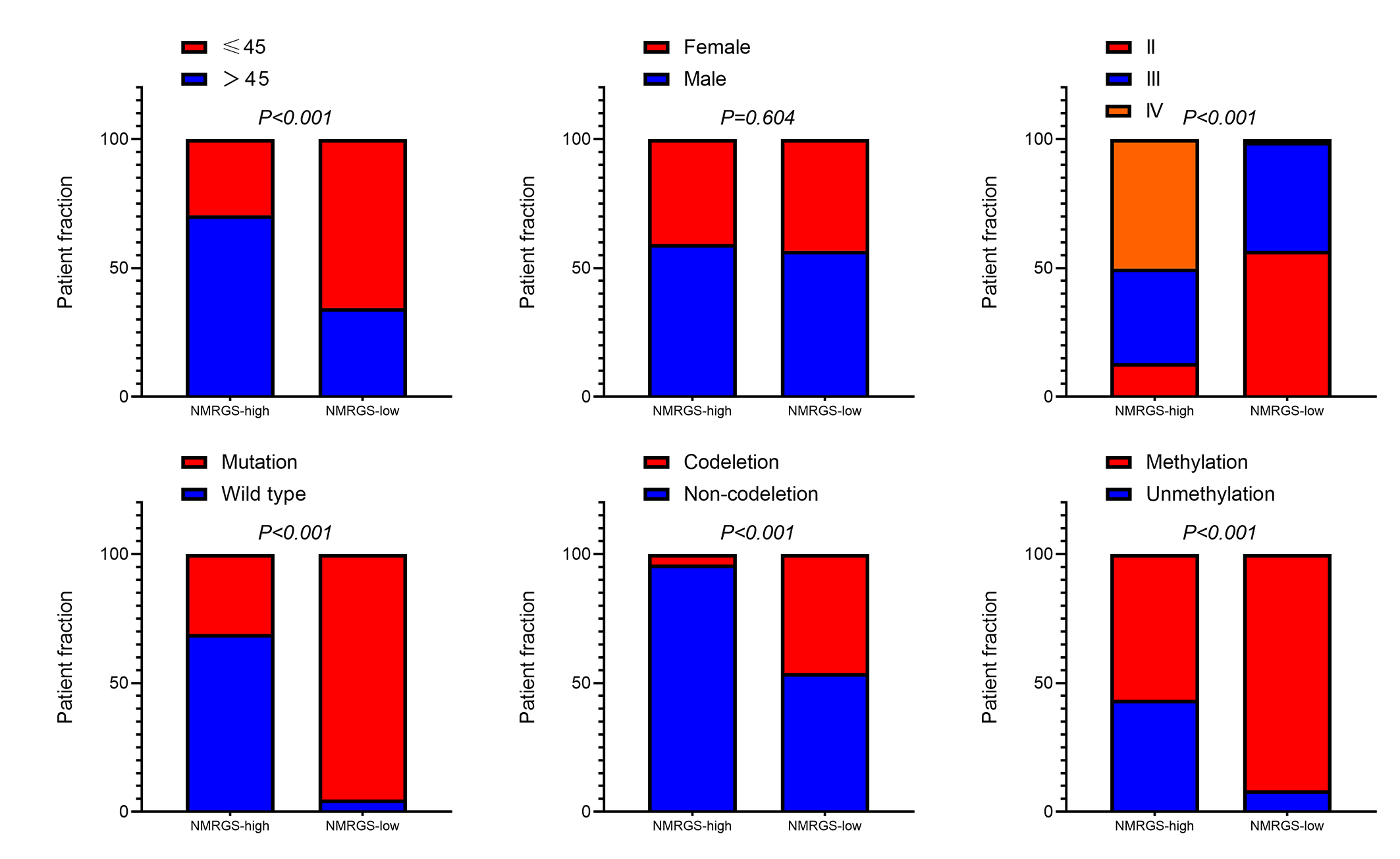

Supplement: Supplementary Figure 9 — Patients fraction in different clinicopathological subgroups in TCGA cohort. [file Image_9.tif]

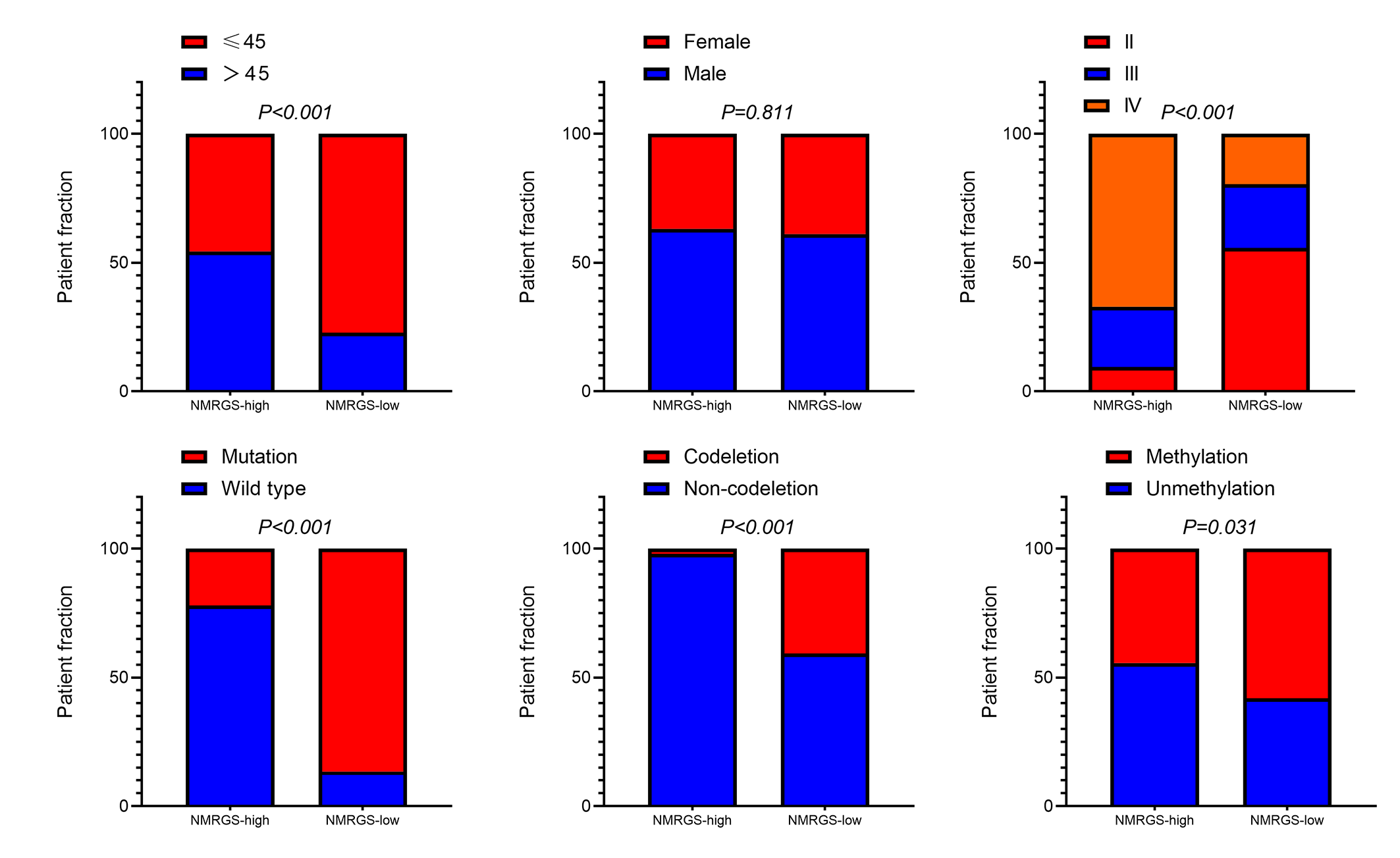

Supplement: Supplementary Figure 10 — Patients fraction in different clinicopathological subgroups in CGGA325 cohort. [file Image_10.tif]

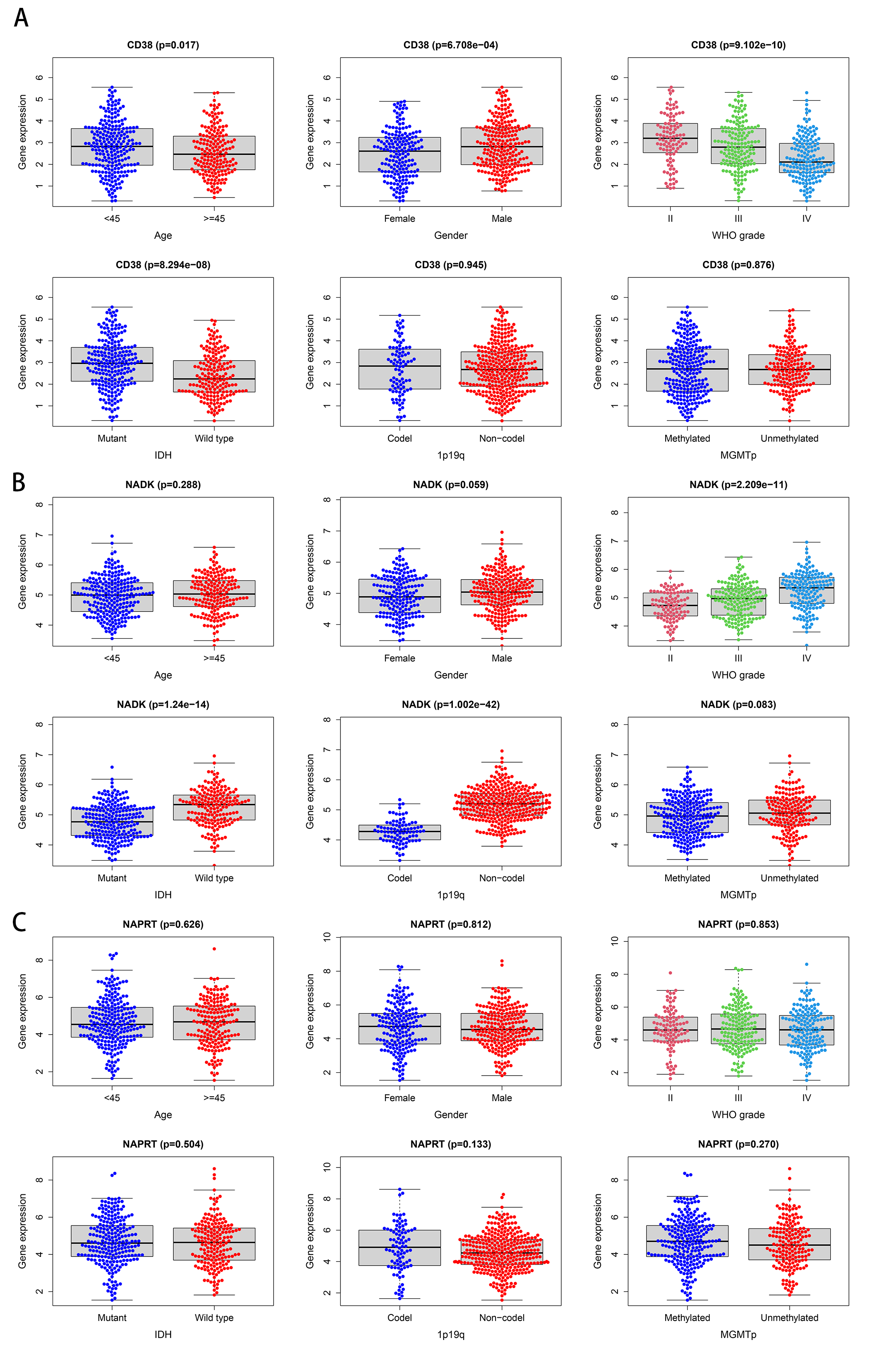

Supplement: Supplementary Figure 11 — Comparison of CD38 (A), NADK (B), NAPRT (C) expression among different clinicopathological subgroups in CGGA693 cohort. [file Image_11.tif]

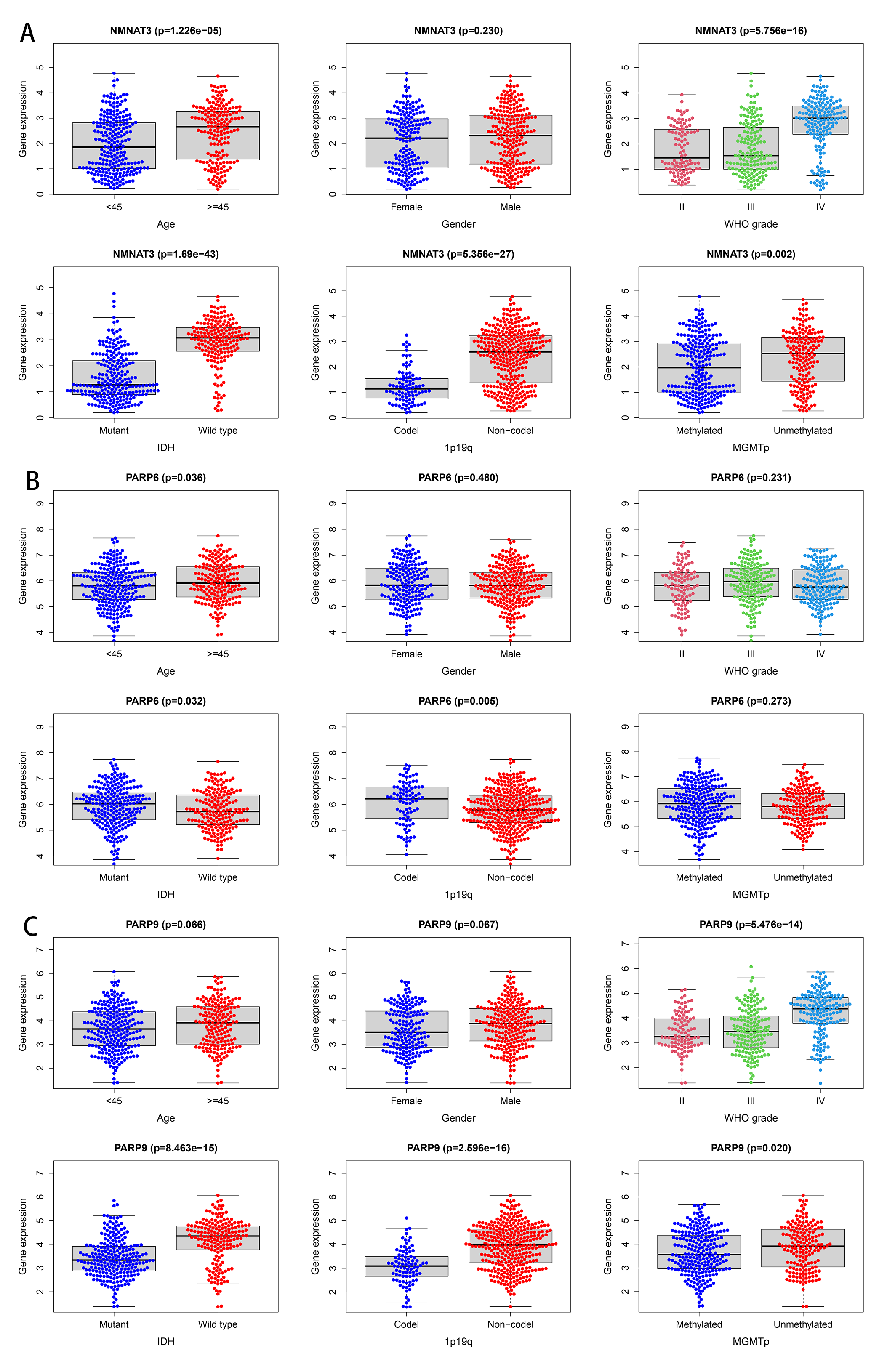

Supplement: Supplementary Figure 12 — Comparison of NMNAT3 (A), PARP6 (B), PARP9 (C) expression among different clinicopathological subgroups in CGGA693 cohort. [file Image_12.tif]

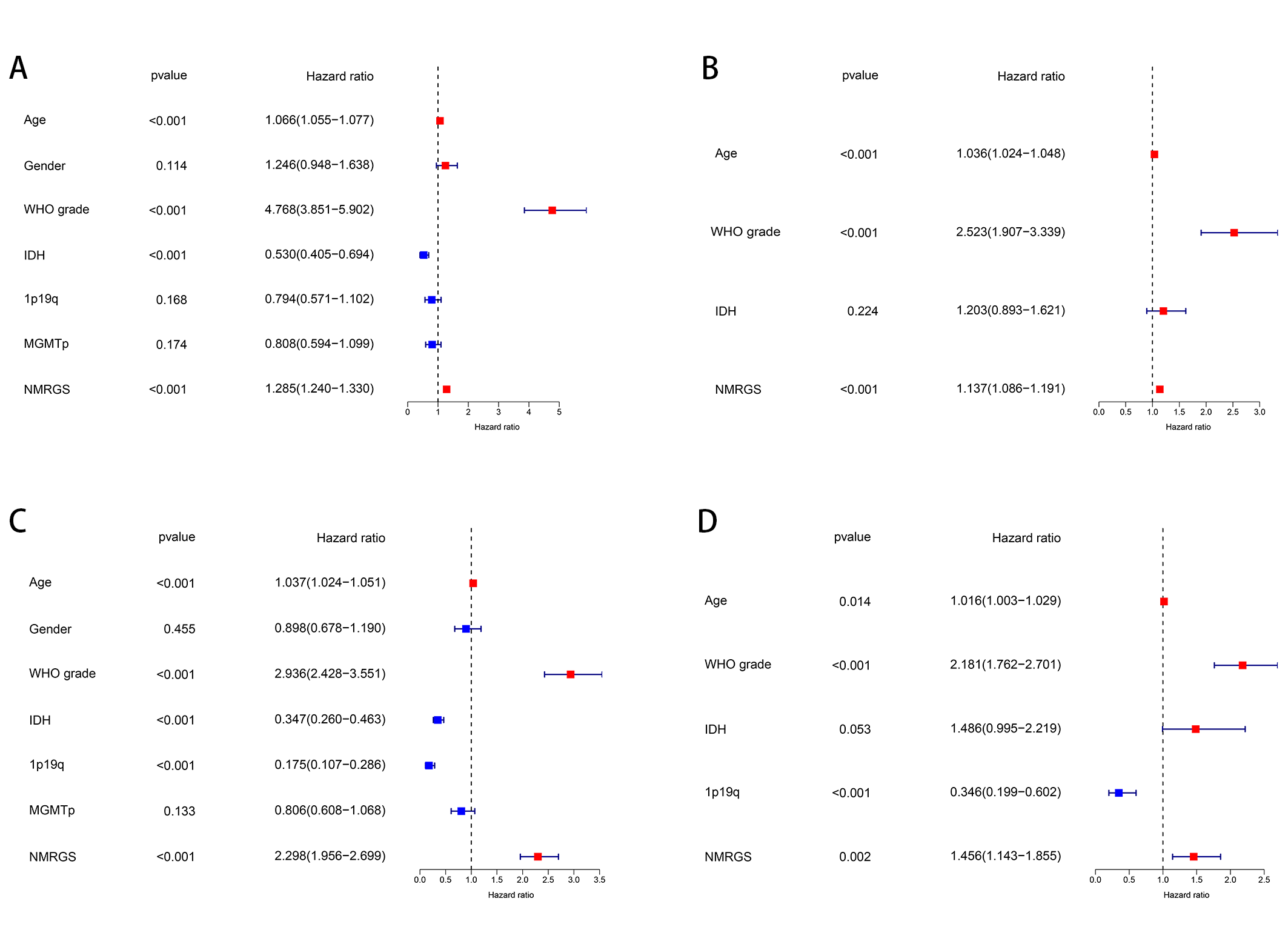

Supplement: Supplementary Figure 13 — (A, B) Univariate and multivariate Cox regression analyses were conducted in TCGA cohort. (C, D) Univariate and multivariate Cox regression analyses conducted in CGGA325 cohort. [file Image_13.tif]

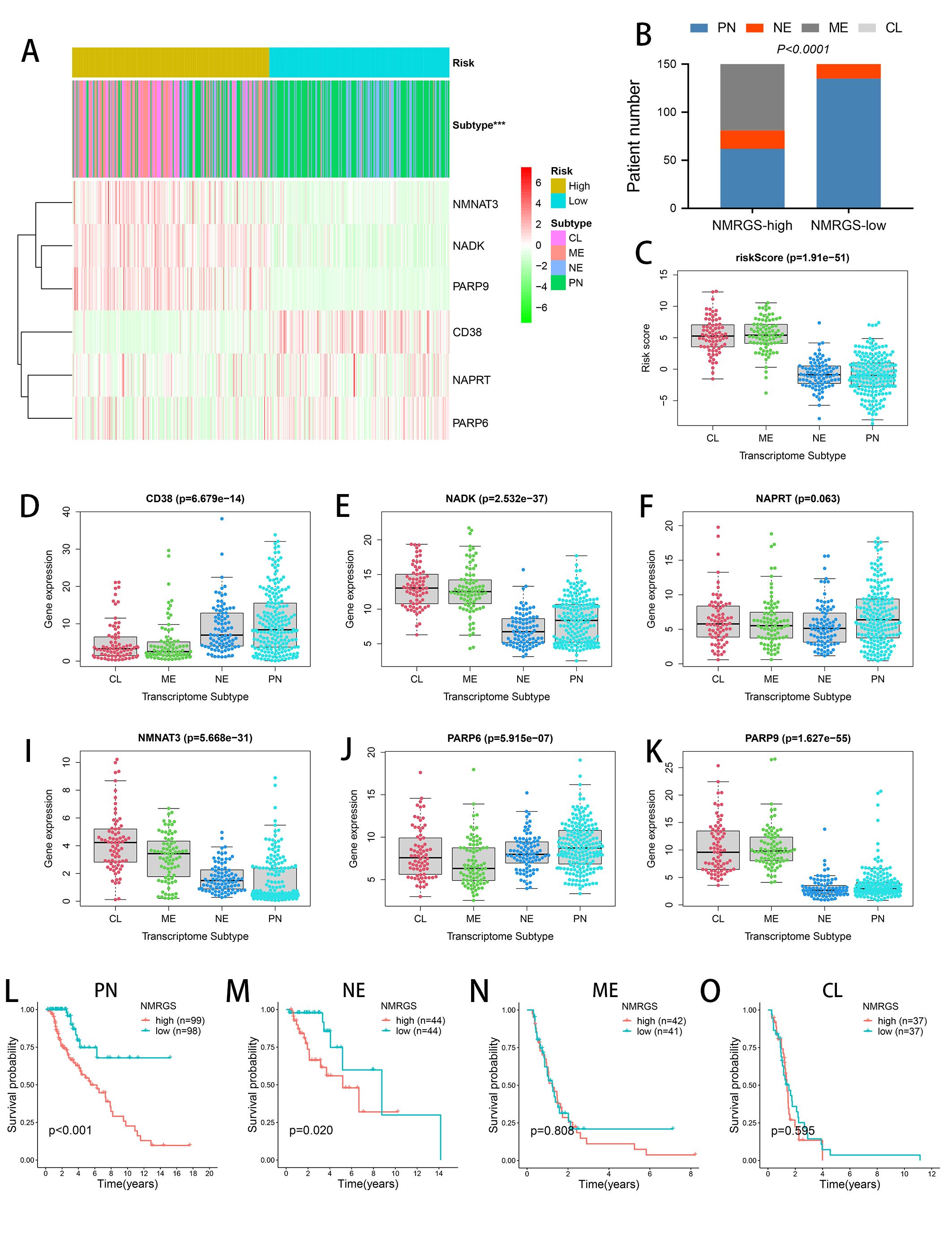

Supplement: Supplementary Figure 14 — The correlation between NMRGS and the four molecular subtypes of glioma. (A) Distribution of the IV subtypes in NMRGS-low group and NMRGS-high group. (B) Patient fraction of these four different subtypes in NMRGS-low group and NMRGS-high group. (C–K) the relationship between the six individual genes and molecular classification. (L–O) KM analysis in different subgroups based on molecular classification. (PN, proneural subgroup; NE, neural subgroup; ME, mesenchymal subgroup; CL, classical subgroup). [file Image_14.tif]

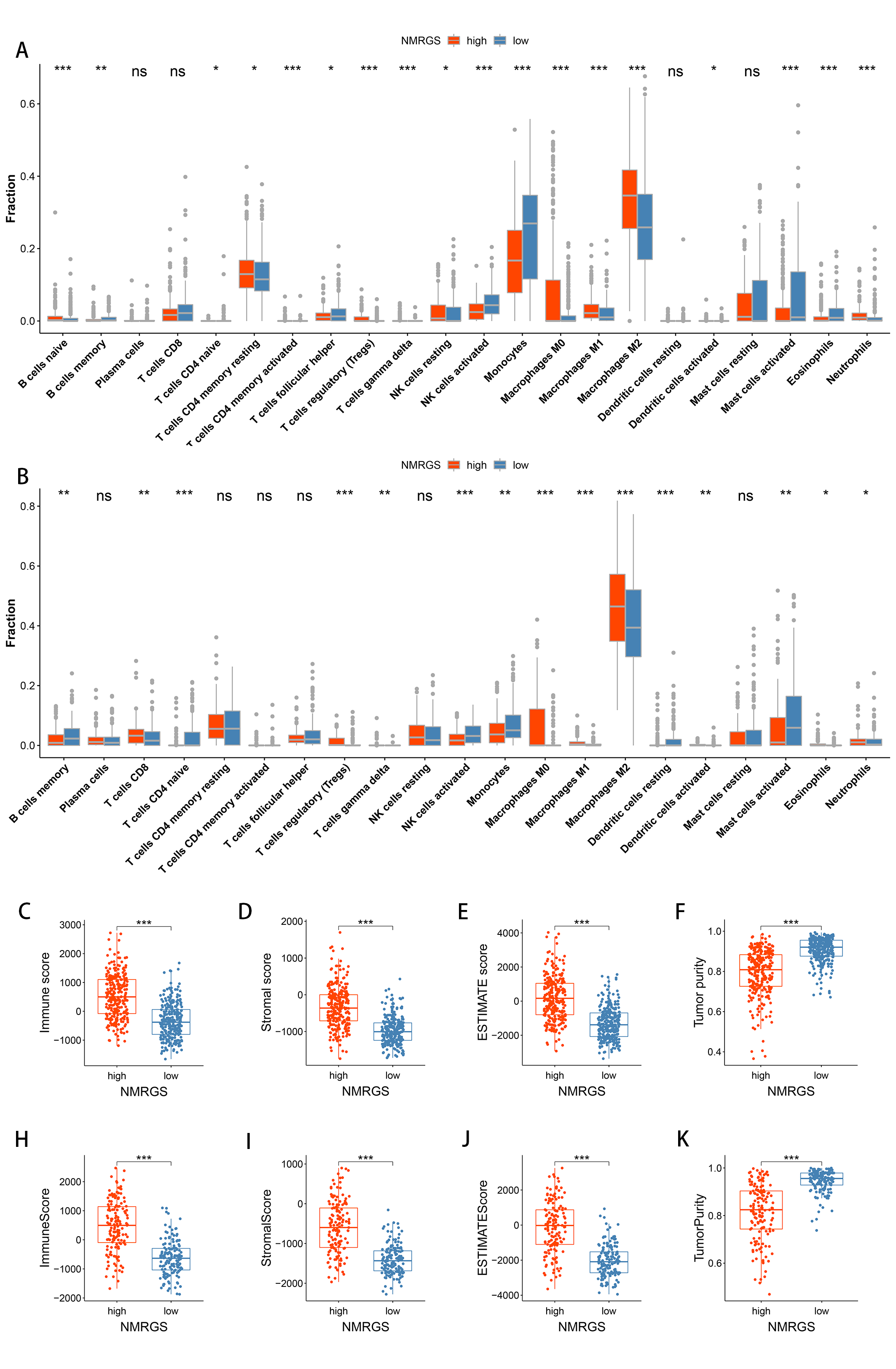

Supplement: Supplementary Figure 15 — (A, B) Comparison of immune cell infiltration between NMRGS-low group and NMRGS-high group in TCGA and CGGA325 cohorts. (C–F) Comparison of ImmuneScore, StromalScore, ESTIMATEScores and TumorPurity between NMRGS-low group and NMRGS-high group in TCGA cohort. (H–K) Comparison of ImmuneScore, StromalScore, ESTIMATEScores and TumorPurity between NMRGS-low group and NMRGS-high group in CGGA325 cohort. [file Image_15.tif]

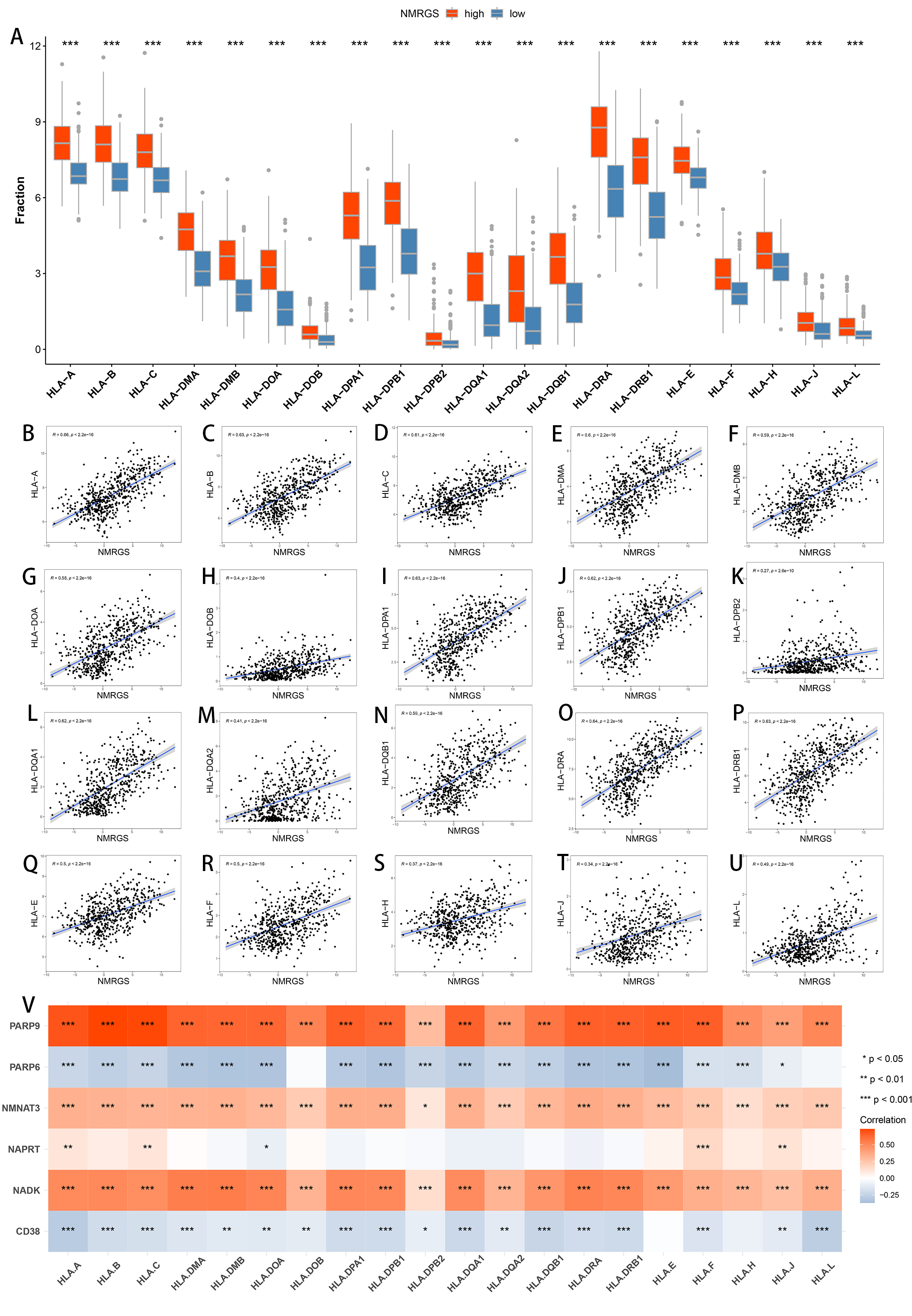

Supplement: Supplementary Figure 16 — Association between NMRGS score and HLA molecules analyzed in TCGA cohort (A) Comparison of HLA molecules between NMRGS-low group and NMRGS-high group. (B–U) Correlations between HLA molecules and the NMRGS score. (V) Correlations between HLA molecules and the six hub NMRGs. [file Image_16.tif]

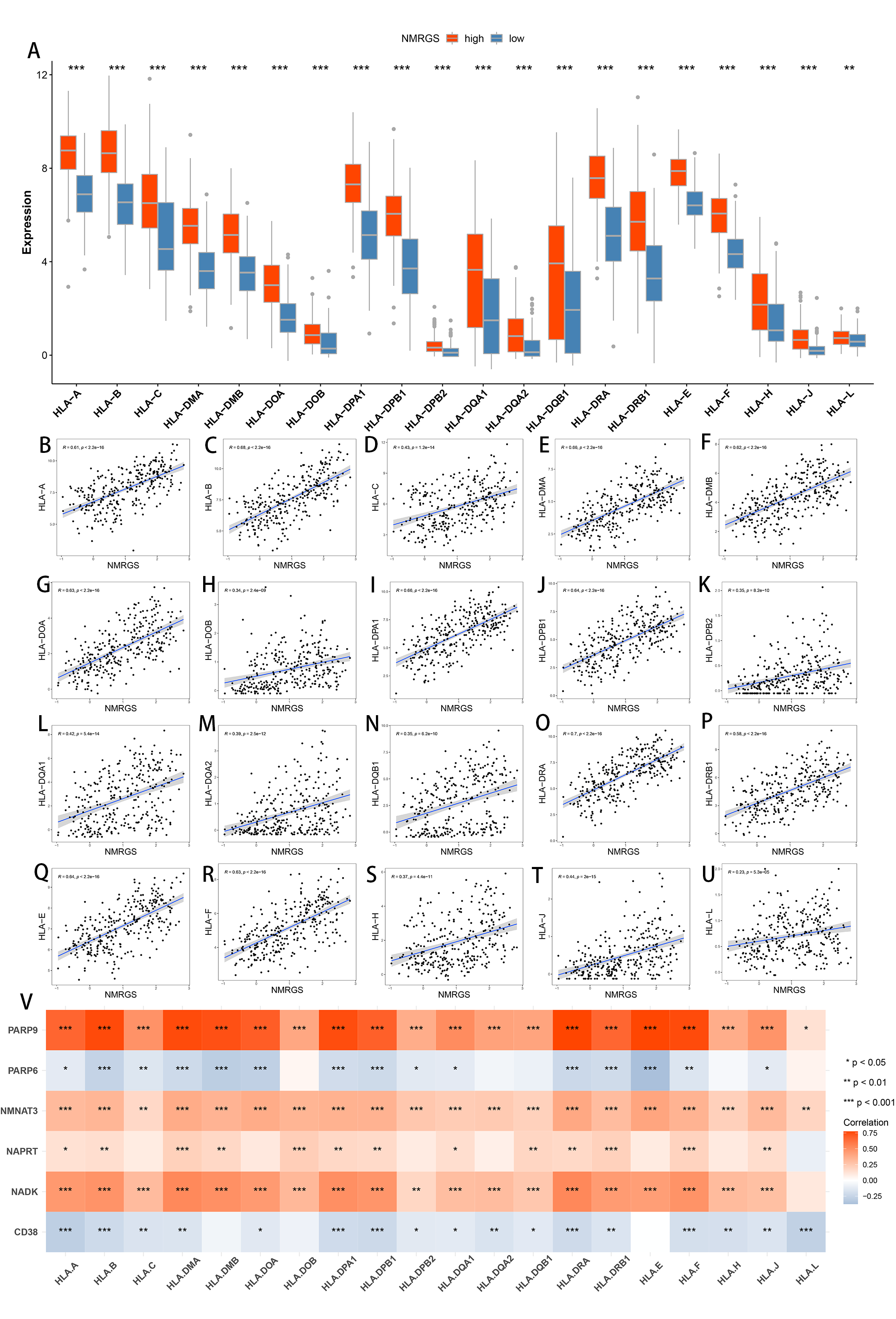

Supplement: Supplementary Figure 17 — Association between NMRGS score and HLA molecules analyzed in CGGA325 cohort (A) Comparison of HLA molecules between NMRGS-low group and NMRGS-high group. (B–U) Correlations between HLA molecules and the NMRGS score. (V) Correlations between HLA molecules and the six hub NMRGs. [file Image_17.tif]

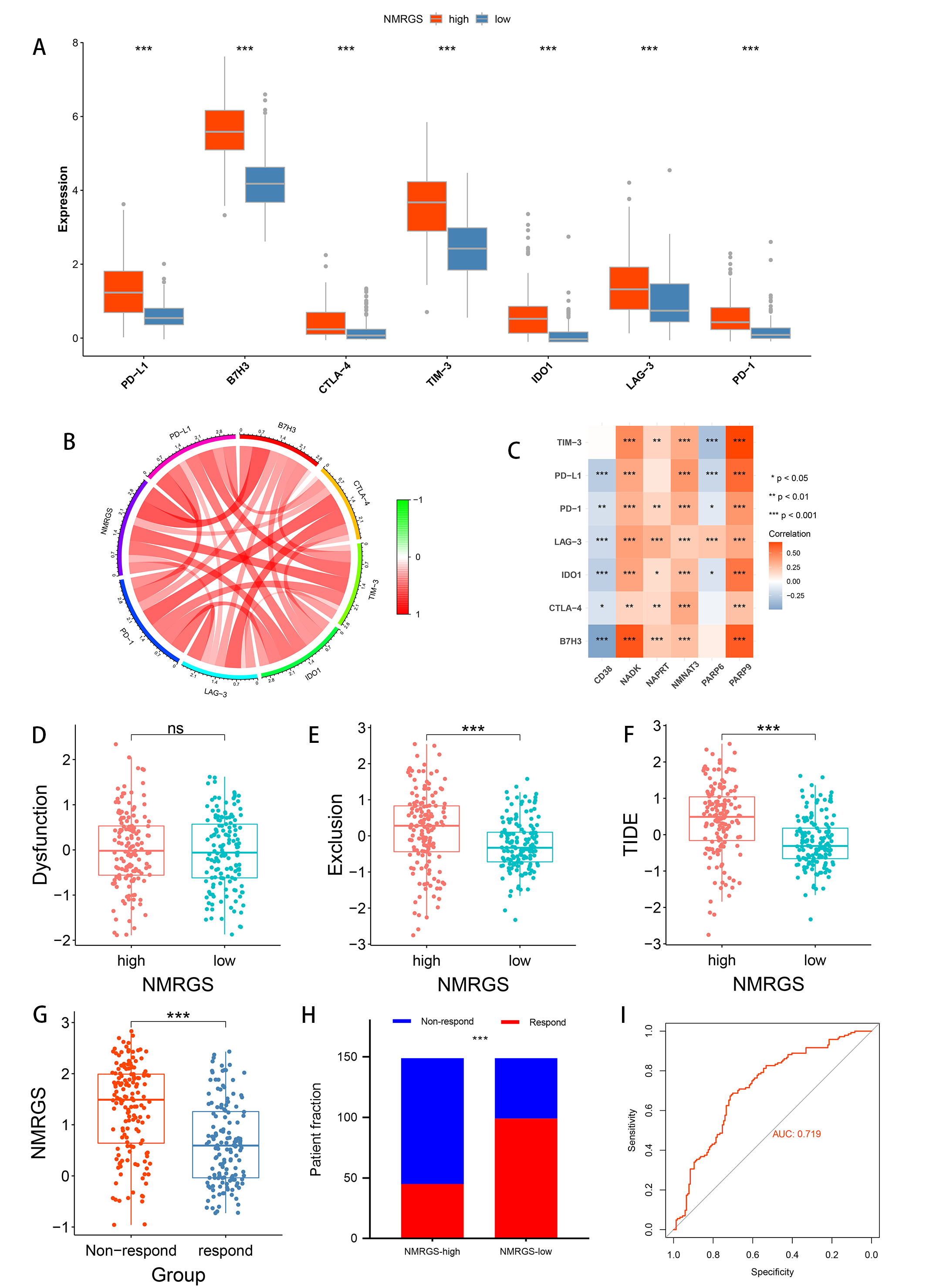

Supplement: Supplementary Figure 18 — Association between NMRGS score and ICI therapy response analyzed in CGGA325 cohort. (A) Comparison of representative immune checkpoints between NMRGS-low group and NMRGS-high group in CGGA325 cohort. (A) Analysis of correlation between NMRGS and representative immune checkpoints expression in CGGA325 cohort. (A) Analysis of correlation between the hub genes and representative immune checkpoints expression in CGGA325 cohort. (D–F) Comparison of dysfunction score, exclusion score and TIDE score between NMRGS-low group and NMRGS-high group in CGGA325 cohort. (G) Comparison of NMRGS score between responders and non-responders in CGGA325 cohort. (H) Patient fraction of responders and non-responders in NMRGS-low group and NMRGS-high group. (I) ROC curve analysis of NMRGS in predicting the efficacy of ICI treatment analyzed in CGGA325 cohort. [file Image_18.tif]

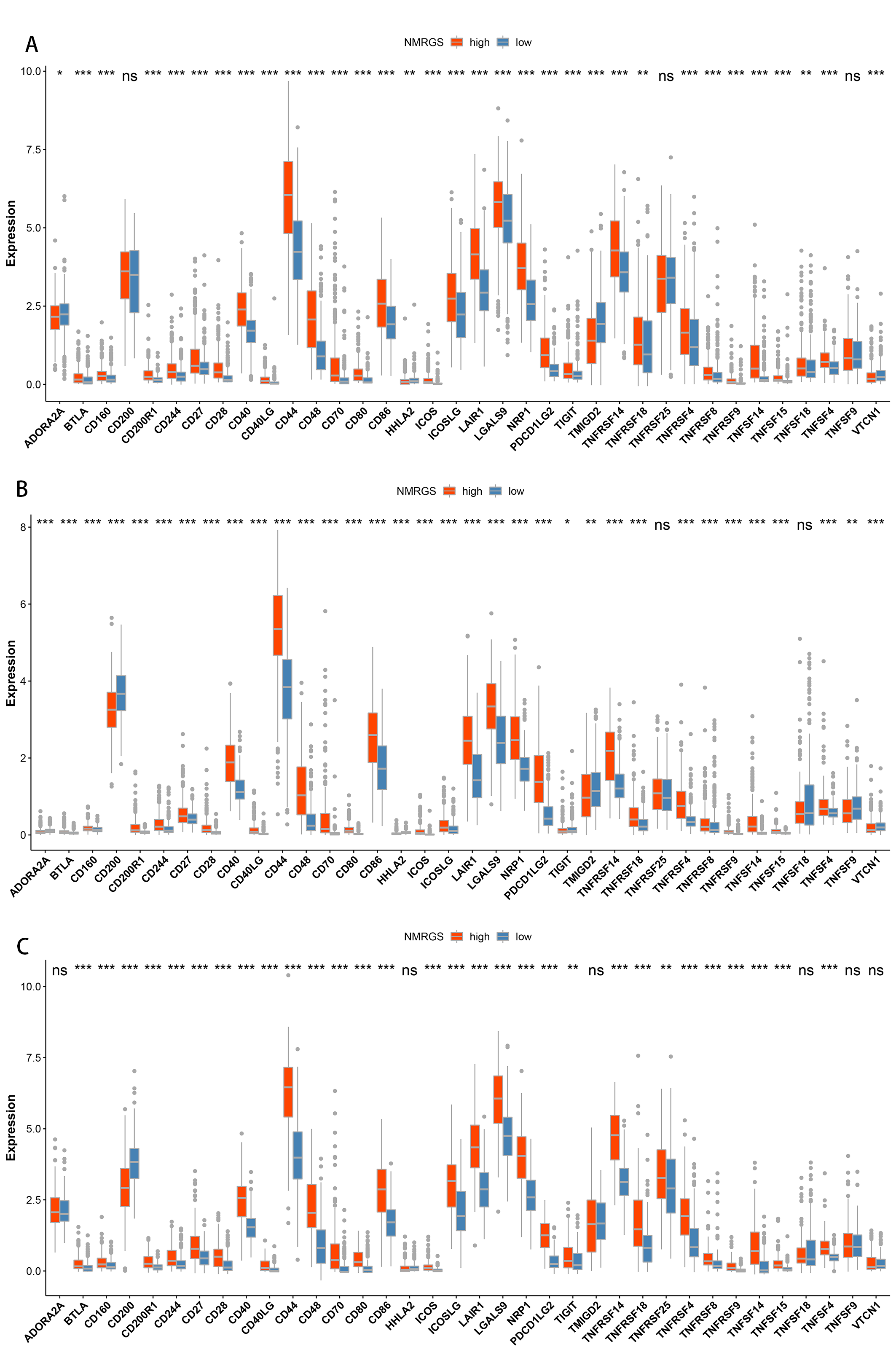

Supplement: Supplementary Figure 19 — Comparison of other immune checkpoints expression between NMRGS-low group and NMRGS-high group in CGGA693, TCGA, and CGGA325 cohorts. [file Image_19.tif]

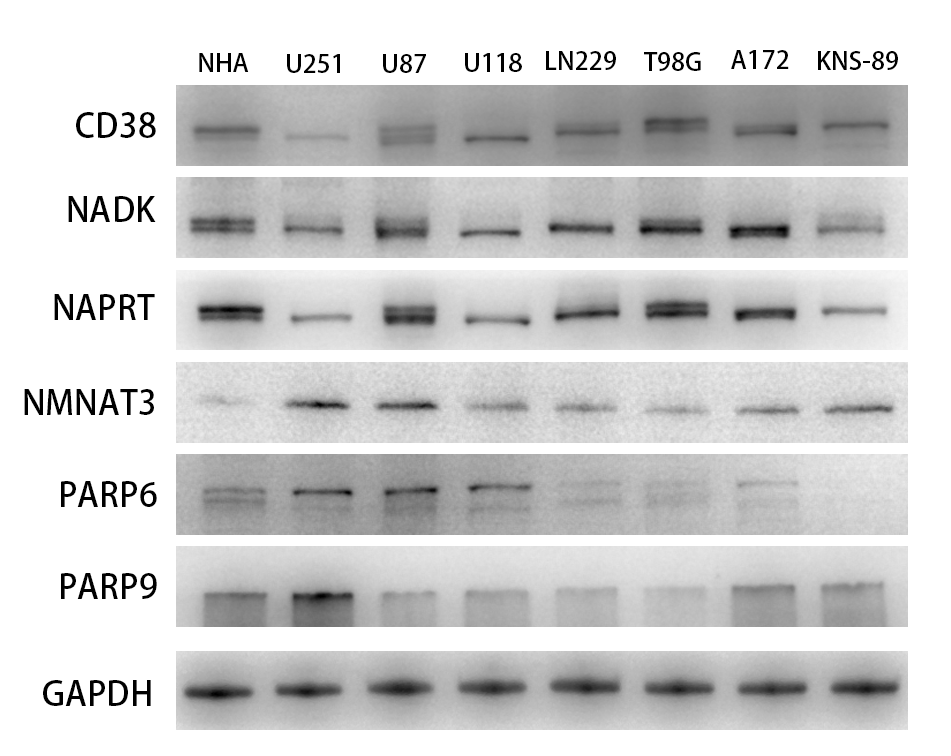

Supplement: Supplementary Figure 20 — Western blots of the hub genes in cell lines. [file Image_20.tif]
