# Supplementary material for: A prognostic NAD+ metabolism-related gene signature for predicting response to immune checkpoint inhibitor in glioma
Source: Front Oncol. 2023 Feb 8;13:1051641. doi: 10.3389/fonc.2023.1051641 (PMC9945104; doi:10.3389/fonc.2023.1051641)
Supplement: Supplementary file 21 [file Table_1.docx]

|  | **Level** | **Overall** | **CGGA693** | **TCGA** | **CGGA325** |
| --- | --- | --- | --- | --- | --- |
| **n** |  | 1486 | 638 | 550 | 298 |
| **futime (mean (SD))** |  | 3.22 (3.01) | 3.38 (2.72) | 2.53 (2.45) | 4.14 (4.04) |
| **fustat (%)** | 0 | 689 (46.4) | 263 (41.2) | 331 (60.2) | 95 (31.9) |
|  | 1 | 797 (53.6) | 375 (58.8) | 219 (39.8) | 203 (68.1) |
| **Gender (%)** | Female | 615 (41.4) | 271 (42.5) | 231 (42.0) | 113 (37.9) |
|  | Male | 871 (58.6) | 367 (57.5) | 319 (58.0) | 185 (62.1) |
| **Age (%)** | <=45 | 830 (55.9) | 386 (60.6) | 261 (47.5) | 183 (61.4) |
|  | >45 | 655 (44.1) | 251 (39.4) | 289 (52.5) | 115 (38.6) |
| **Grade (%)** | WHO II | 460 (31.0) | 171 (26.8) | 192 (34.9) | 97 (32.6) |
|  | WHO III | 533 (35.9) | 244 (38.2) | 217 (39.5) | 72 (24.2) |
|  | WHO IV | 493 (33.2) | 223 (35.0) | 141 (25.6) | 129 (43.3) |
| **IDH status (%)** | Mutant | 834 (58.4) | 329 (55.8) | 344 (63.5) | 161 (54.2) |
|  | Wildtype | 595 (41.6) | 261 (44.2) | 198 (36.5) | 136 (45.8) |
| **1p19q status (%)** | Codel | 337 (23.9) | 137 (23.9) | 138 (25.4) | 62 (21.2) |
|  | Non-codel | 1074 (76.1) | 437 (76.1) | 406 (74.6) | 231 (78.8) |
| **MGMT promoter status (%)** | Methylated | 827 (63.3) | 293 (57.9) | 391 (75.0) | 143 (51.1) |
|  | Unmethylated | 480 (36.7) | 213 (42.1) | 130 (25.0) | 137 (48.9) |

**Supplementary Table S1: Clinical information of glioma patients from the CGGA693, TCGA, CGGA325 cohorts.**
