# Supplementary material for: A prognostic NAD+ metabolism-related gene signature for predicting response to immune checkpoint inhibitor in glioma
Source: Front Oncol. 2023 Feb 8;13:1051641. doi: 10.3389/fonc.2023.1051641 (PMC9945104; doi:10.3389/fonc.2023.1051641)
Supplement: Supplementary file 22 [file Table_2.docx]

**Supplementary Table S2. Metabolism-related genes (NMRGs)**

| NMRGs | Ensembl ID | Full name |
| --- | --- | --- |
| AOX1 | ENSG00000138356 | Aldehyde Oxidase 1 |
| BST1 | ENSG00000109743 | Bone Marrow Stromal Cell Antigen 1 |
| CD38 | ENSG00000004468 | CD38 Molecule |
| CYP8B1 | ENSG00000180432 | Cytochrome P450 Family 8 Subfamily B Member 1 |
| ENPP1 | ENSG00000197594 | Ectonucleotide Pyrophosphatase/Phosphodiesterase 1 |
| ENPP3 | ENSG00000154269 | Ectonucleotide Pyrophosphatase/Phosphodiesterase 3 |
| NADK | ENSG00000008130 | NAD Kinase |
| NADK2 | ENSG00000152620 | NAD Kinase 2, Mitochondrial |
| NADSYN1 | ENSG00000172890 | NAD Synthetase 1 |
| NAMPT | ENSG00000105835 | Nicotinamide Phosphoribosyltransferase |
| NAPRT | ENSG00000147813 | Nicotinate Phosphoribosyltransferase |
| NAXD | ENSG00000213995 | NAD(P)HX Dehydratase |
| NAXE | ENSG00000163382 | NAD(P)HX Epimerase |
| NMNAT1 | ENSG00000173614 | Nicotinamide Nucleotide Adenylyltransferase 1 |
| NMNAT2 | ENSG00000157064 | Nicotinamide Nucleotide Adenylyltransferase 2 |
| NMNAT3 | ENSG00000163864 | Nicotinamide Nucleotide Adenylyltransferase 3 |
| NMRK1 | ENSG00000106733 | Nicotinamide Riboside Kinase 1 |
| NMRK2 | ENSG00000077009 | Nicotinamide Riboside Kinase 2 |
| NNMT | ENSG00000166741 | Nicotinamide N-Methyltransferase |
| NNT | ENSG00000112992 | Nicotinamide Nucleotide Transhydrogenase |
| NT5C | ENSG00000125458 | 5', 3'-Nucleotidase, Cytosolic |
| NT5C1A | ENSG00000116981 | 5'-Nucleotidase, Cytosolic IA |
| NT5C2 | ENSG00000076685 | 5'-Nucleotidase, Cytosolic II |
| NT5C3A | ENSG00000122643 | 5'-Nucleotidase, Cytosolic IIIA |
| NT5E | ENSG00000135318 | 5'-Nucleotidase Ecto |
| NT5M | ENSG00000205309 | 5',3'-Nucleotidase, Mitochondrial |
| NUDT12 | ENSG00000112874 | Nudix Hydrolase 12 |
| PARP10 | ENSG00000178685 | Poly(ADP-Ribose) Polymerase Family Member 10 |
| PARP14 | ENSG00000173193 | Poly(ADP-Ribose) Polymerase Family Member 14 |
| PARP16 | ENSG00000138617 | Poly(ADP-Ribose) Polymerase Family Member 16 |
| PARP4 | ENSG00000102699 | Poly(ADP-Ribose) Polymerase Family Member 4 |
| PARP6 | ENSG00000137817 | Poly(ADP-Ribose) Polymerase Family Member 6 |
| PARP8 | ENSG00000151883 | Poly(ADP-Ribose) Polymerase Family Member 8 |
| PARP9 | ENSG00000138496 | Poly(ADP-Ribose) Polymerase Family Member 9 |
| PNP | ENSG00000198805 | Purine Nucleoside Phosphorylase |
| PTGIS | ENSG00000124212 | Prostaglandin I2 Synthase |
| PTGS2 | ENSG00000073756 | Prostaglandin-Endoperoxide Synthase 2 |
| QPRT | ENSG00000103485 | Quinolinate Phosphoribosyltransferase |
| RNLS | ENSG00000184719 | Renalase, FAD Dependent Amine Oxidase |
| SLC22A13 | ENSG00000172940 | Solute Carrier Family 22 Member 13 |
