# Supplementary material for: A prognostic NAD+ metabolism-related gene signature for predicting response to immune checkpoint inhibitor in glioma
Source: Front Oncol. 2023 Feb 8;13:1051641. doi: 10.3389/fonc.2023.1051641 (PMC9945104; doi:10.3389/fonc.2023.1051641)
Supplement: Supplementary file 23 [file Table_3.docx]

**Supplementary Table S3. The Primers sequence used in this study**

| **Name** | **Forward-primer** | **Reverse-primer** |
| --- | --- | --- |
| CD38 | CCAAAGTGTATGGGATGC | CATTAGTGGCTGATAGTCTTC |
| NADK | GGCTGCTTACTGCTGCTC | TCCTGAACTCCTTGGTGC |
| NAPRT | CTGGAGTCAGTCCTCATCGTA | GTCAGCTTCATTCGTGGC |
| NMNAT3 | GACCCAAAAGGTTACATCG | GACAGCATCGGGAATCAG |
| PARP6 | GCCTCAGCATCTTTTCAC | ACTTCCACTTTGGGGTTC |
| PARP9 | CGGTGGATGGAATGGGATA | AGGCTGGAATTGCTACTGTCTT |
