# Supplementary material for: A prognostic NAD+ metabolism-related gene signature for predicting response to immune checkpoint inhibitor in glioma
Source: Front Oncol. 2023 Feb 8;13:1051641. doi: 10.3389/fonc.2023.1051641 (PMC9945104; doi:10.3389/fonc.2023.1051641)
Supplement: Supplementary file 24 [file Table_4.docx]

**Supplementary Table S4. The primary antibodies used in immunohistochemistry**

| **Protein** | **Antibody Name** | **Catalogue Number** | **Company** | **Antibody Concentration** |
| --- | --- | --- | --- | --- |
| CD38 | CD38 Polyclonal antibody | 25284-1-AP | Proteintech | 1:200 |
| NADK | NADK Polyclonal antibody | 15548-1-AP | Proteintech | 1:200 |
| NAPRT | NAPRT Polyclonal antibody | 13549-1-AP | Proteintech | 1:200 |
| NMNAT3 | NMNAT3 Polyclonal antibody | 206497-T08 | Sino Biological | 1:100 |
| PARP6 | PARP6 Polyclonal antibody | DF13800 | Affinity Biosciences | 1:100 |
| PARP9 | PARP9 Polyclonal antibody | 17535-1-AP | Proteintech | 1:200 |
