# Supplementary material for: A prognostic NAD+ metabolism-related gene signature for predicting response to immune checkpoint inhibitor in glioma
Source: Front Oncol. 2023 Feb 8;13:1051641. doi: 10.3389/fonc.2023.1051641 (PMC9945104; doi:10.3389/fonc.2023.1051641)
Supplement: Supplementary file 25 [file Table_5.docx]

**Supplementary Table S5. Patient information for IHC**

| **Patient** | **Gender** | **Age** | **Pathological Information** | **WHO Grade** | **Tissue** |
| --- | --- | --- | --- | --- | --- |
| Patient 1 | Male | 61 | glioblastoma multiforme | Ⅳ | TT |
| Patient 2 | Female | 45 | Diffuse Astrocytoma | Ⅱ | TT and NBT |
| Patient 3 | Male | 52 | glioblastoma multiforme | Ⅳ | TT |
| Patient 4 | Female | 70 | glioblastoma multiforme | Ⅳ | TT |
| Patient 5 | Male | 56 | glioblastoma multiforme | Ⅳ | TT |
| Patient 6 | Male | 47 | Anaplastic oligodendroglioma | Ⅲ | TT |
| Patient 7 | Female | 58 | glioblastoma multiforme | Ⅳ | TT and NBT |
| Patient 8 | Male | 54 | ganglioglioma | Ⅱ | TT |
| Patient 9 | Male | 58 | anaplastic astrocytoma | Ⅲ | TT and NBT |
| Patient 10 | Male | 38 | Diffuse Astrocytoma | Ⅱ | TT and NBT |
| Patient 11 | Female | 56 | glioblastoma multiforme | Ⅳ | TT |
| Patient 12 | Male | 45 | glioblastoma multiforme | Ⅳ | TT and NBT |

TT: Tumor tissue; NBT: normal brain tissue
